# Metabolomics of *Myrcia bella* Populations in Brazilian Savanna Reveals Strong Influence of Environmental Factors on Its Specialized Metabolism

**DOI:** 10.3390/molecules25122954

**Published:** 2020-06-26

**Authors:** Luiz Leonardo Saldanha, Pierre-Marie Allard, Adlin Afzan, Fernanda Pereira de Souza Rosa de Melo, Laurence Marcourt, Emerson Ferreira Queiroz, Wagner Vilegas, Cláudia Maria Furlan, Anne Lígia Dokkedal, Jean-Luc Wolfender

**Affiliations:** 1 Faculty of Sciences, São Paulo State University (UNESP), CEP 17033-360, Bauru, São Paulo, Brazil; fernandamelo405@gmail.com (F.P.d.S.R.d.M.); dokkedal@fc.unesp.br (A.L.D.); 2 School of Pharmaceutical Sciences, Institute of Pharmaceutical Sciences of Western Switzerland, University of Geneva (IPSWS), CH-1211 Geneva 4, Switzerland; pierre-marie.allard@unige.ch (P.-M.A.); Adlin.Afzan@unige.ch (A.A.); laurence.marcourt@unige.ch (L.M.); Emerson.Ferreira@unige.ch (E.F.Q.); 3 Institute of Biosciences, São Paulo State University (UNESP), CEP 11330-900, São Vicente, São Paulo, Brazil; vilegasw@gmail.com; 4 Institute of Biosciences, University of São Paulo, CEP 05508-090, São Paulo, São Paulo, Brazil; furlancm@ib.usp.br

**Keywords:** *Myrcia bella*, Myrtaceae, Brazilian savanna, phytogeographic patterns, metabolomics

## Abstract

Environmental conditions influence specialized plant metabolism. However, many studies aiming to understand these modulations have been conducted with model plants and/or under controlled conditions, thus not reflecting the complex interaction between plants and environment. To fully grasp these interactions, we investigated the specialized metabolism and genetic diversity of a native plant in its natural environment. We chose *Myrcia bella* due to its medicinal interest and occurrence in Brazilian savanna regions with diverse climate and soil conditions. An LC-HRMS-based metabolomics approach was applied to analyze 271 samples harvested across seven regions during the dry and rainy season. Genetic diversity was assessed in a subset of 40 samples using amplified fragment length polymorphism. Meteorological factors including rainfall, temperature, radiation, humidity, and soil nutrient and mineral composition were recorded in each region and correlated with chemical variation through multivariate analysis (MVDA). Marker compounds were selected using a statistically informed molecular network and annotated by dereplication against an in silico database of natural products. The integrated results evidenced different chemotypes, with variation in flavonoid and tannin content mainly linked to soil conditions. Different levels of genetic diversity and distance of populations were found to be correlated with the identified chemotypes. These observations and the proposed analytical workflow contribute to the global understanding of the impact of abiotic factors and genotype on the accumulation of given metabolites and, therefore, could be valuable to guide further medicinal exploration of native species.

## 1. Introduction

*Myrcia bella* Cambess (Myrtaceae) is popularly known as “mercurinho”. Is an important and common plant native to the Brazilian Savanna (Cerrado) [1]. In Brazil, populations of *M. bella* are widely distributed in different regions of the Cerrado Domain. Leaves of this plant are used in traditional medicine to treat gastrointestinal disorders and diabetes [2]. Pharmacological studies have demonstrated the cytotoxicity [2], antimicrobial [3], and antidiabetic [4] properties of the hydroalcoholic extracts of its leaves. In all these studies, phenolic compounds were related to bioactivity. Phytochemical studies of the leaves of *M. bella* have described several derivatives of O-glycoside flavonols and phenolic acids [3,5,6].

Plant metabolites play important ecophysiological roles in response to the environment [7]. Biotic and abiotic factors affect the biosynthesis of a wide range of specialized metabolites [8,9,10]. Variation in the production of metabolites has been observed not only among different species, but also between specimens of the same species growing under different environmental conditions. Specific environmental factors have been identified as the main source of intra-species metabolism variations. For example, abiotic environmental conditions such as soil nutrients and water availability can induce the accumulation of specific compounds in different parts of plants growing in different localities [11,12]. For medicinal exploitation purposes, it is therefore important to identify the factors (biotic, abiotic, and seasonal) that can affect the production and accumulation of specialized metabolites in populations of species.

Untargeted metabolite analysis (metabolomics) has been successfully used for the comprehensive analysis of a wide range of small molecules produced by a given organism, providing functional information in response to biotic or abiotic stress [13,14]. When performed on carefully selected biological replicates, it can provide information about changes at the chemical level of species or specimens related to genetic variability, abiotic stress, and/or interaction with other organisms [15]. Most comparative metabolomics studies have been carried out on model crop plants subjected to controlled environmental conditions to demonstrate relationships between plant metabolites, genotypes, and phenotypes [16,17] and to elucidate biological processes [18].

However, experiments involving model crops and controlled conditions only approximate the condition of plants in their natural environment. Plants possess natural genetic variability and are constantly submitted to concurrent environmental factors which reveal their full phenotypic plasticity. For example, Sampaio et al. [11] used untargeted metabolomics to demonstrate the effects of abiotic environmental factors in situ on *Tithonia diversifolia* (Helms.) A. Gray, (Asteraceae) using clonal specimens. Therefore, direct monitoring of metabolic changes under natural conditions is needed to better understand the interrelationship between the environment and the plant metabolome.

As metabolomics studies generate large and complex datasets, multivariate statistical data analysis (MVDA) is required to extract relevant information. MVDA gives statistical values for the metabolites and their correlation with the analyzed factors of interest. Dereplication is then performed to identify known metabolites from spectral or structural features. Recently, molecular network (MN) analysis integration with in silico spectral databases (ISDB) has been proposed to annotate structurally related metabolites [19]. The generated MN is composed of clusters of nodes grouped based on the similarity of their fragmentation patterns under identical ionization conditions. Since the development of MN analysis, several strategies have been developed to integrate biological and chemical information, facilitating the process of bioactive compound identification [20]. For example, Saesong et al. combined bioactivity results and taxonomic information to identify clusters of active metabolites within extracts of the medicinal plants of the genus *Bacopa* (Plantaginaceae) [21]. Olivon et al. expanded these approaches by merging different bioassay data from hundreds of Euphorbiaceae extracts, generating a massive multi-informative MN to guide the isolation of compounds of interest [22]. Given the importance of MN in the discovery of bioactive compounds, many of these prior studies have naturally focused on bioactivity-based approaches. Likewise, given that most comparative metabolomics studies have focused on model crop plants in controlled conditions or using genetically homogeneous specimens, there is a need for metabolomics studies of non-model plants under natural conditions to determine the factors affecting plant metabolism and assess metabolic changes.

In order to address this issue, we selected *M. bella* for its importance in traditional medicine as a model to perform metabolomics of specimens distributed in different regions of Brazilian Cerrado and exhibiting characteristic microclimate and soil variations. The Cerrado climate is defined by two well-marked seasons, namely the dry season and the rainy season, which has higher temperatures [23]. Soils typically have low fertility, are acidic, and have high levels of aluminum [24].

Our goal was to investigate metabolite content and genetic fingerprints among plant populations of *M. bella* growing under a wide range of environmental conditions and to determine the abiotic environmental factors that putatively affect its specialized metabolism. Analysis of 271 samples collected in rainy and dry seasons over a 24 month period was performed with an optimized ultra-high performance liquid chromatography and high-resolution mass spectrometry UHPLC-HRMS method. Metabolite profiles and environmental factors were correlated using MVDA methods. A MN was created after HRMS2 analysis for extensive metabolite annotation in comparison with in silico fragmentation spectral database. The metabolomics data were treated through standard MVDA and, when such statistical results were integrated in a multi-informational molecular networks, this approach highlighted reliable and important changes in metabolite families.

## 2. Results

The sampling plan implemented in this study allowed the collection of leaf samples from 271 specimens of *Myrcia bella* from seven different regions from the Brazilian savanna (Table 1) during both dry and rainy seasons over 24 months (Tables S1, S2, Figure S1).

**Figure 1:**
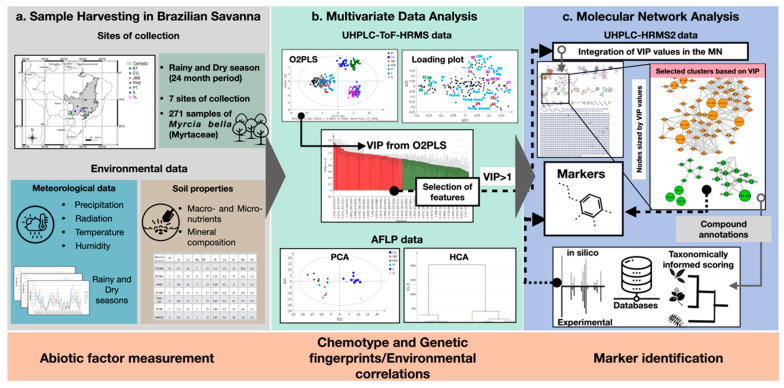
Summary of the workflow used in this study. (**a**) Strategy followed for sample harvesting and environmental monitoring in different regions of the Brazilian savanna. (**b**) Multivariate data analysis was used to analyze the chemical and genetic data and to correlate compounds with significant variation (markers) to environmental factors. (**c**) A multi-informative molecular network was then generated by merging metabolomics multivariate data (VIP values) in the molecular network to identify chemotype markers. Compounds were annotated by spectral matching against in silico fragmentation databases, following a taxonomically informed reranking process.

The workflow established for the analysis and data mining is summarized in Figure 1 and detailed hereafter.

All samples were profiled by UHPLC-ToF-HRMS to generate metabolomics data that were processed by MVDA to differentiate groups of specimens and identify chemotype markers. Such MS data were correlated with meteorological and soil properties information to highlight specific environmental factors that could possibly be involved in metabolite profile variations. In parallel, for a selected subset of 40 representative samples, DNA AFLP marker analysis allowed the genetic diversity and distance among populations to be evaluated.

For metabolite identification, a mix of all samples was analyzed by complementary UHPLC-HRMS/MS and data-dependent acquisition analysis on the largest number of detected features. The MS2 dataset was organized using the Global Natural Products Social Network platform (GNPS) to generate a MN of all detected features. These compounds were annotated based on their MS2 spectra compared with experimental or in silico MS/MS databases and identified by authentic standards. The main markers were highlighted in the MN based on their VIP values related to group differentiation after MVDA analysis.

### 2.1. Data Acquisition and Processing for Multivariate Analysis

A chromatographic gradient was developed to enable the analysis of a high number of samples in a unique batch while ensuring a satisfactory resolution and a low ion-suppression level. The metabolite fingerprinting was performed in negative ion mode to highlight phenolic compounds that are known to be present in *Myrcia bella* [5].

As an effort to eliminate bias due to potential gradual changes on the UHPLC-ToF-HRMS platform performance, the test samples were randomized and quality control (QC) samples were injected every 20 analyses to validate the quality of the profiles obtained [25,26]. Thus, based on the QC sample validation, the variability of retention time, mass accuracy, and area intensities in chromatograms were within acceptable ranges and indicated the instrument stability over circa 72 h (Figure S2).

### 2.2. Multivariate Data Analysis

Principal component analysis (PCA) was performed using Pareto and log-transformed, and showed better clustering of samples compared with the non-log transformed PCA (Figure S3). Nine principal components were calculated by cross-validation, and the first two components (PCs) explained 27.3% and 0.9% of the variation in the spectral data, respectively. The PCA prediction was validated based on R2x (0.585) and Q2 (0.432), which were in an acceptable range for biological a PCA model [26]. An acceptable level of data quality was also indicated in this study by QC samples (Figure S3) tightly clustered and near the plot origin. The analysis of the distribution of samples in PCA revealed a few outliers outside of the model boundary (Hotelling’s T2 of 95%) (data not shown).

The PCA (Figure S3) and hierarchical cluster analysis HCA dendrogram (Figure S4) results provided good discrimination of all samples and clearly showed the presence of three main groups. As evidenced, specimens from the populations of the Goiás and São Paulo regions, represented by the Parque Nacional das Emas (PNE), Jardim Botânico Municipal de Bauru (JBB), and Pratânia (PT), were clustered together and were named chemotype CI. Meanwhile, specimens from the Mato Grosso do Sul regions were clustered in two main groups, one represented by the Bonito (BT) and Campo Grande (CG) populations, named chemotype CII, and other formed by Selvíria (S) and Três Lagoas (TL), named chemotype CIII. This grouping pattern was consistent with the geographical position of the areas of sampling. However, Goiás and São Paulo populations, which were distant from each other, shared the same chemotype. Nevertheless, PCA analysis of the soil data (Figure S5) from the seven localities revealed that these two sites possessed similar soil properties.

Possible metabolomics variations between the rainy and dry periods were investigated, creating separate PCA models for each population (Figure S6); however, no grouping related to dry or rainy seasons was observed.

Altogether, the PCA and HCA results provided a good discrimination of all samples and clearly evidenced the presence of three chemotypes of *M. bella* across the studied regions. Furthermore, our results suggest that the chemical variability resulting in the three chemotypes (CI, CII, and CIII) could be mainly linked to specific soil and meteorological conditions, and to a lesser extent to water availability. These correlations are further investigated and discussed below.

### 2.3. Identification of Site-Specific Environmental Factors from Supervised Analysis Results

**Figure 2:**
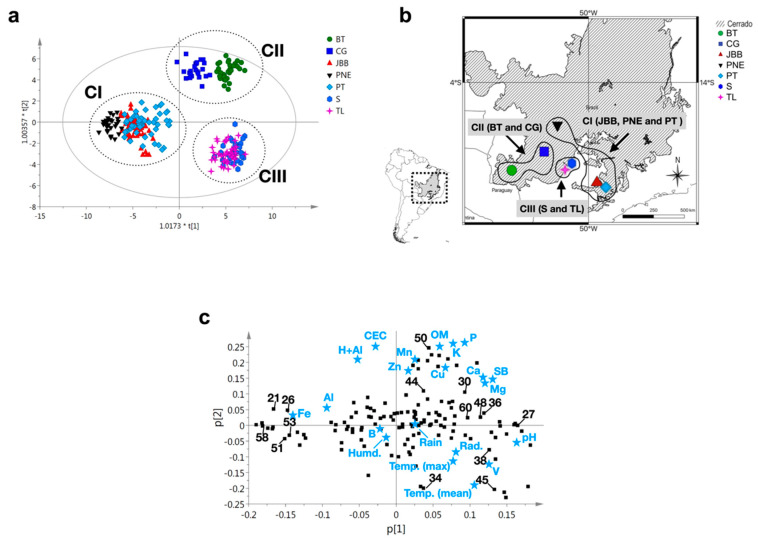
Multivariate data analysis of UHPLC-ToF-HRMS fingerprinting data of 271 *Myrcia bella* extracts collected in seven regions of Cerrado. (**a**) O2PLS score plot highlighting the identified chemotypes (CI, CII, and CIII). (**b**) Geographical map summarizing the location of the harvesting site. The black lines delineate regions sharing the same chemotype. (**c**) O2PLS loading plot exhibiting the correlation of the environmental factors with given metabolites (numbers in black). List of abbreviations: Fe = soil iron; Al = soil aluminum; Mn = soil manganese; K = soil potassium; Cu = soil copper; P = soil phosphorus; Mg = soil magnesium; Zn = soil zinc; Ca = soil calcium; SB = soil sum of basis; pH = soil pH; V = soil bases saturation; CEC = cation exchange capacity; Temp. (mean) = mean air temperature; Temp. (max) = maximum air temperature. BT = Bonito; CG = Campo Grande; S = Selvíria; TL = Três Lagoas; PNE = Parque Nacional das Emas; PT = Pratânia; JBB = Jardim Botânico de Bauru. GO = Goiás; SP = São Paulo; MS = Mato Grosso do Sul.

The two-way orthogonal PLS (O2PLS) model [27], which is used to analyze the relationships between two different matrices (X and Y), was created to identify the environmental factors possibly responsible for inducing chemical variations (Figure 2a).

This model allowed us to correlate the metabolomics data (302 features as X input) with meteorology and soil factors (22 factors as Y input) coherently. The O2PLS model was then constructed using 271 observations with a good fit and predictivity (R2Ycum was 0.758 and Q2 cum was 0.713). This model showed very little orthogonal variation explained at 9%, which could be attributed to intra-population variation. The O2PLS analysis showed clear separation between all samples, evidencing the three chemotypes previously observed in the PCA analysis (Figure 2a). The groupings produced are mapped in Figure 2b.

As shown in the loading plot from the O2PLS (Figure 2c), the edaphic conditions as the micronutrients iron (Fe) and exchangeable aluminum (Al), potential acidity (H+Al), and cation exchange capacity (CEC), contributed negatively to the PC1, and were correlated to the Goiás (PNE) and São Paulo (JBB and PT) regions. On the other hand, the levels of macronutrients (Mg, Ca, P, and K) and micronutrients (Cu and Mn), besides soil properties such as the sum of bases (SB) and organic matter (OM) level, were positively correlated to the Mato Grosso do Sul regions of BT and CG. Other environmental conditions such as thermal amplitudes (mean, maximum, and minimum temperatures), radiation incidence, and soil properties such as pH as well as base saturation (V), were correlated to the S and TL (Mato Grosso do Sul) regions.

Besides humidity, rain, and boron (B), all the other factors analyzed in the study were correlated to specific localities, which was consistent with our findings, since no difference was detected on the metabolome of *M. bella* extracts between dry and rainy seasons.

### 2.4. Molecular Networking Analysis

In order to obtain structural information of features, MS/MS spectra of the QC (pool of all extracts) and authentic reference compounds were recorded by UHPLC-Orbitrap. The preliminary MS data treatment yielded 1296 features with associated MS2 data in negative-ion mode. These were organized using the GNPS platform to generate a unique molecular network (MN) using the feature-based molecular networking workflow (FBMN) [28]. In this MN, the nodes corresponding to each feature were grouped into 396 clusters according to their fragmentation patterns’ similarity. By comparing the retention order and exact masses, features could be correlated between the ToF/MS fingerprints (MVDA) and the Orbitrap profiles (MN and metabolite annotation analysis).

### 2.5. Metabolite Annotation

In a second step, the annotation of compounds in the MN was performed at Level 2 [29]. The acquired MS2 spectra of each node from the whole MN were searched automatically against an in silico theoretical spectra database built from the Dictionary of Natural Products (ISDB-DNP) following a previously described methodology [19]. Subsequently, a taxonomically informed metabolite annotation strategy was applied [30]. For this, the initial ISDB-DNP output annotations ranked according to their spectral similarity were re-ranked based on a score attributed to candidates for which the biological source was found to be Myrtaceae at the family level, *Myrcia* at the genus level, and/or *M. bella* at the species level. Using this approach, among the 1296 features, 545 (42%) compounds were annotated in the MN. The identities of ten annotated compounds were confirmed at Level 1 [29] by co-injection of authentic standards isolated from *M. bella* [5] and MS/MS spectra comparison (Figure S7).

### 2.6. Integration of Multivariate Data Analysis with Molecular Networking for the Identification of Chemotype Markers

**Figure 3:**
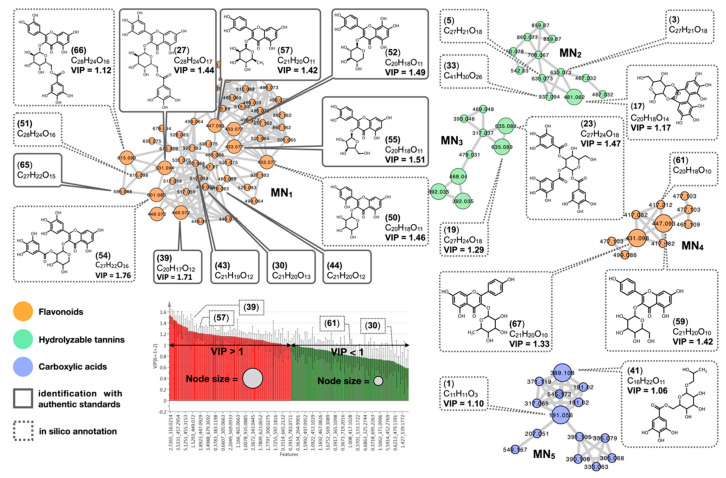
Selected clusters (MN_1_–MN_5_) from the statistically informed molecular network. VIP values greater than 1 (represented in red in the VIP plot) from the O2PLS model were integrated into the molecular network and can be visualized through the node size. Larger nodes indicate features with VIP values greater than 1. Dotted line boxes indicate putatively annotated compounds (ISDB-DNP in silico annotations), and full lines indicate dereplicated compounds for which identity was confirmed by comparing the spectroscopic data with compounds isolated from *Myrcia bella*. Green, orange, or purple colors indicate the chemical classes of the compounds. Flavonoid, tannin, and carboxylic acid chemical class clusters with representative structures are depicted.

In an attempt to assemble both MVDA from fingerprinting and feature annotations by MN, the VIP of significant features was linked as metadata to create a statistically informed MN (Figure S8). For this, 63 features with a VIP value > 1.0 in the O2PLS analysis were visualized through the size of the node in the MN (Figure 3). Such features were displayed in the MN in larger size nodes. Nodes with VIP values below 1 or without statistical data available were kept at their original size.

Using this approach, we were able to highlight clusters of families of compounds related to statistically relevant features spotted through the MVDA. This allowed us to both add statistically relevant information to the MN, and to demonstrate spectral similarity among previously unrelated MVDA features.

After a visual inspection of the statistically informed MN, clusters were selected based on their node size (higher nodes indicate VIP > 1.0). This resulted in the selection of five clusters, herein named MN_1_–MN_5_ (Figure 3). A total of 78 compounds were annotated among the selected clusters (Table S4). Of those, 39 compounds were identified as flavonoids, 17 as carboxylic acid derivatives, 19 as hydrolyzable tannins, and 3 as chromones.

The correlations between these compounds and the environmental factors of each geographic region are presented in the loading plot (Figure 2c). Accordingly, the flavonoids 21, 26, 51, 53, and 58 were mainly found in plant populations collected from the Goiás (PNE) and São Paulo (JBB and PT) regions (chemotype CI). In these regions, the soil shows higher levels of iron and aluminum, and a low pH (pH ~ 4).

On the other hand, the flavonoids 27, 30, 36, 44, and 50, the carboxylic acid 48, and chromone 60 were related to plant specimens found in the Mato Grosso do Sul region (CG and BT) (chemotype CII). In this region, the soil shows higher levels of copper, calcium, manganese, potassium, calcium, magnesium, and zinc, and low potential base saturation (V). Finally, the hydrolyzable tannin 34, the carboxylic acid 38, and chromone 45 were related to specimens collected in Mato Grosso do Sul regions (S and TL) (chemotype CIII), with a high influence of environmental factors such as radiation, temperature ranges (maximum and mean), and higher pH (~5).

To further explore the correlation between the level of content of these compounds in all collected *M. bella* specimens and the environmental factors studied, a correlation analysis was performed (Figure S9). Compounds with significant correlation (*p* < 0.001) to given environmental factors are highlighted in the correlation matrix. This analysis confirmed that compounds 21, 26, 47, 52, 58, 65, 67, 74, 75, 76, and 77 were positively correlated with low pH and high Fe and Al levels. Compounds 17, 30, 25, 27, and 37 were positively correlated with K, Ca, Mg, Cu, and SB, and compounds 1, 14, 27, 37, and 45 were positively correlated with higher temperature. These correlations (Figure S8) confirmed the results discussed above (Figure 2c).

In addition, we analyzed the variable plot line displaying the level of compounds across samples (Figure S10). Accordingly, compounds 21, 26, and 47 were found exclusively in chemotype CI, represented by specimens from São Paulo (JBB and PT) and Goiás (PNE) regions. On the other hand, compounds 1, 14, 37, 50, and 54 were found in higher levels in both CII and CIII chemotypes represented by specimens from all Mato Grosso do Sul (BT, CG, S, and TL) regions. This suggests that the three chemotypes of *M. bella* mainly differ in their contents of flavonoids, carboxylic acids, and hydrolyzable tannins. Since these compounds were found exclusively or in higher levels in specific regions, they were selected as markers to distinguish the three chemotypes.

### 2.7. Genetic Diversity Based on Amplified Fragment Length Polymorphism (AFLP)

The genetic diversity of a selected subset of 40 representative samples from the six localities was assessed through amplification of restriction fragments from total digest of genomic DNA (Figure S11). The AFLP method is a DNA fingerprinting technique suited for applications in genetic analysis such as genetic relationship and diversity assessments [31]. In this study, the AFLP analysis was performed using four combinations of EcoRI and MseI primers with three selective nucleotides each. Each combination gives different patterns of amplified fragments. The primers Eco-ACC/MseI-CAT yielded 291 alleles, Eco-ACG/MseI-CAG yielded 196 alleles, Eco-ACC/MseI-CAA yielded 316 alleles, and Eco-ACG/MseI-CTG yielded 225 alleles. In total, 1028 loci using four combinations of EcoRI and MseI primers with three selective nucleotides each were analyzed.

**Figure 4:**
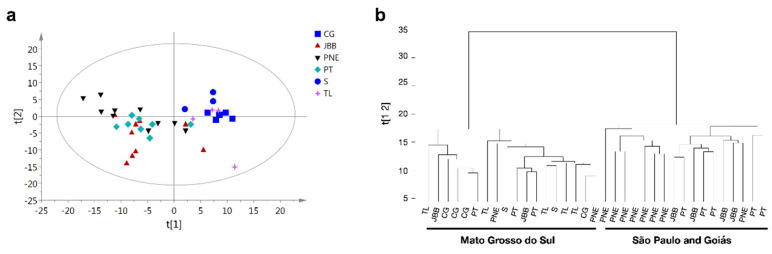
Multivariate data analysis of the AFLP markers data of *Myrcia bella* samples from different regions of Cerrado. (**a**) PCA score plot derived from 40 samples. (**b**) HCA plot derived from the generated PCA score plot. Both PCA and HCA highlighted the presence of two main genotype clusters. List of abbreviations: CG = Campo Grande; S = Selvíria; TL = Três Lagoas; PNE = Parque Nacional das Emas; PT = Pratânia; JBB = Jardim Botânico de Bauru.

As for the observations on the PCA and HCA analysis of the AFLP data (Figure 4), two main clusters were observed according to their localities. Specimens from São Paulo (JBB and PT) and Goiás (PNE) were grouped together, while specimens from Mato Grosso do Sul (CG, S, and TL) formed another group.

Parameters of intrapopulation genetic diversity, including the percentage of polymorphic loci (P) and genetic diversity (He), are presented in Table 2.

The results showed that the percentage of polymorphic bands per population (P) ranged from 11.58% to 54.77%. The populations from Parque Nacional das Emas (PNE), Goiás region, Jardim Botânico de Bauru (JBB), and Pratânia (PT) from the São Paulo region presented higher values of percentage of polymorphic bands, while Três Lagoas (TL), Campo Grande (CG), and Selvíria (S), from the Mato Grosso do Sul region, presented lower values.

Intrapopulation genetic diversity (He) ranged from 0.048 to 0.135 based on AFLP analysis. Selvíria (S), Campo Grande (CG), and Três Lagoas (TL) populations from the Mato Grosso do Sul region presented lower values than other populations of Jardim Botânico de Bauru (JBB) and Pratânia (PT) from the São Paulo region, and of Parque Nacional das Emas (PNE) from the Goiás region.

The genetic distance between populations [32,33] assessed using the unweighted pair group method with arithmetic mean (UPGMA) is presented in Table 3.

The analysis revealed that specimens from Jardim Botânico de Bauru were significantly close to Pratânia (0.026) and to Parque Nacional das Emas (0.022), and more similar to Três Lagoas (0.078), while Selvíria was the most genetically distant from the other populations studied. No correlations between genetic and geographical distances were found.

## 3. Discussion

Based on the metabolic differences detected and the environmental factors analyzed, as well as literature data, it was possible to interpret the main sources of chemical variations among the populations of *M. bella* studied in the Cerrado Domain. Our results revealed that seasonal changes in precipitation between dry and rainy seasons were not as significant as soil conditions, temperature ranges, and solar radiation.

The analysis of the seven populations collected in specific regions revealed the existence of three *M. bella* chemotypes (Figure 2a,b). These three chemotypes were found to exhibit strong links with the soil properties of the location where specimens were sampled.

The *M. bella* specimens (three populations, JBB, PT, and PNE) collected in the Sāo Paulo and Goiás regions were found to belong to the same chemotype (chemotype CI). These two regions have in common high levels of soil aluminum and iron. On the other hand, the *M. bella* specimens collected (four populations, BT, CG, S, and TL) in the Mato Grosso do Sul region formed two main chemotypes, assigned as chemotype CII (CG, BT) and chemotype CIII (S and TL). In the Mato Grosso do Sul regions of CG and BT, the soil exhibited high levels of copper, phosphorus, calcium, zinc, manganese, magnesium, organic matter, and sum of bases, while the S and TL regions exhibited higher temperature and pH as well as of solar radiation levels compared to all other regions.

The chemotypes evidenced (CI, CII, and CIII) and the environmental correlations found in our study indicated that specific environmental factors act on *M. bella*’s metabolism. The sources of chemical variation were different at each of the studied areas.

Plant–soil interactions have been described by van Nuland et al. [34] as an important ecological and evolutionary process associated with changes in plant phenotype and fitness, which might ultimately affect genetic divergence among populations, adaptive or contemporary evolution, and diversification [35,36]. Soil gradients and biota may influence the expression and evolution of plant phenotypes [37,38,39].

Environmental factors such as solar radiation and temperature ranges are well described to affect plant metabolism [11,40,41]. Several soil properties and conditions such as pH and organic matter and nutrient availability are able to induce effects on the biosynthesis and accumulation of given metabolites [42,43]. Soil nutrients such as calcium, phosphorus, potassium, manganese, and copper are essential for normal higher plant growth and can be responsible for inducing physiological responses in different plant organs [11,43,44].

In this study, the *M. bella* specimens were harvested in southeastern and southern regions of the Cerrado Domain, a large region that occupies the center of South America with different microclimates and soil compositions [24,45]. Most of the Cerrado Domain areas, which include both forest and savanna habitats in a mosaic-like distribution [46], are on plateaus of crystalline or sedimentary blocks interrupted by inter-plateau depressions, forming geomorphological regions originating from major dynamic changes during the Tertiary and Quaternary periods. Several authors have demonstrated the strong relationship between soil type and vegetation physiognomy and composition [47,48,49]. The soils of Cerrado areas are usually acidic, well drained, deep, and characterized by a low availability of nutrients and toxicity related to high levels of metals such as aluminum, manganese, and iron [24].

Aluminum is the main soil metal inhibiting plant growth and functions in acid soils [50,51,52,53,54,55]. At the cellular level, the strong binding affinity of aluminum with oxygen-donor ligands, such as proteins, nucleic acids, and phospholipids, results in the inhibition of cell division, cell extension, and transport [56].

Our study indicated that specific flavonoids were correlated with high contents of soil metals (iron and aluminum) in the Sāo Paulo (JBB and PT) and Goiás regions (PNE). Other studies have shown that plants can exude flavonoids such as catechin and quercetin to chelate aluminum as a mechanism of tolerance to metal stress [42,52,57]. Carboxylic acids and flavonoids can bind to aluminum to form non-toxic complexes, protecting cell components from oxidative damage caused by high exposure to this metal [52,58,59].

Tannins were found in higher levels and were correlated to high supply of soil nutrients and high contents of manganese and organic matter (OM) in the Mato Grosso do Sul (BT and CG) region. Several studies have demonstrated the influence of manganese in the shikimic acid pathway, resulting in the accumulation of tannins in plants [43,60]. Apart from that, some authors investigated the impact of tannin soil exudate from plant roots on microbial diversity and activity, which are crucial for nutrient availability dynamics from organic matter degradation [61]. Polyphenols, especially tannins, are known to affect either positively or negatively microorganisms’ activities, which in turn influence nitrogen mineralization and nitrification of soil organic matter by several mechanisms [61,62,63]. Tannins may additionally increase nutrient availability (e.g., iron, phosphorus, copper, and manganese) by forming organic complexes, and also retain exchangeable inorganic cations (calcium, potassium, and magnesium) by providing sorption sites in highly acidic soils [64].

In parallel to the results of the metabolomics data, the genetic analysis of the subset of samples from six populations revealed two main genetic clusters based on their polymorphisms (Figure 4a,b, Table 2, Table 3). The genetic distance analysis showed that *M. bella* specimens from São Paulo (JBB and PT) and Goiás (PNE) (chemotype CI) had high genetic similarity and were distant from the Mato Grosso do Sul populations. These specimens belonging to chemotypes CII and CIII were genetically close to each other and distant to chemotype CI.

The analysis of intrapopulation genetic diversity revealed that populations from Goiás and Sāo Paulo (chemotype CI) presented the highest genetic diversity. These specimens were collected from conservation areas such the Jardim Botânico de Bauru (forest) and Parque Nacional das Emas (national park). Other studies using the AFLP technique in protected areas with native species from Brazilian savanna have found similar levels of intrapopulation genetic diversity [65,66,67,68]. Lower values of intrapopulation genetic diversity were found for Campo Grande, Três Lagoas, and Selvíria populations (chemotypes CII and CII) from areas under anthropic pressure. As described by Schlaepfer et al. [69], the increased inbreeding and gene flow caused by anthropogenic fragmentation of natural areas may reduce the genetic variation of remnant populations.

Altogether, the metabolomics and genetics results evidenced the presence of three chemotypes and two genetic clusters among the regions studied. Populations with higher values of genetic diversity (PNE, JBB, and PT) formed one chemotype, while low genetic diversity (CG, S, and TL) resulted in two chemotypes.

From the genetic point of view, in areas with higher anthropic pressure, the populations, even those with lower genetic diversity, showed greater chemical diversity, suggesting a greater investment in chemical defense. As expected, populations in areas with lower anthropic pressure showed high genetic diversity.

Through this study, our results clearly indicated that stressful abiotic soil properties and temperature conditions, together with anthropogenic pressure, drive chemical and genetic variability among populations of *M. bella* and define their phenotypes.

These functional differences in terms of metabolism were found to be relatively well correlated with the genetic analysis of populations. However, more in-depth studies with a larger number of samples in these regions are necessary for a better understanding of the relationship between chemical and genetic diversity in different environments.

From an ecological perspective, the results obtained in our investigation showed also a great level of consistency with previous floristic studies [46,48], revealing that edaphic conditions have influence not only in the metabolome at the species level, but also on vegetation type and composition at the landscape level.

It should be mentioned that previous bioactivity studies [2,3,4,6] have indicated that specific flavonoids are most likely involved in the medicinal properties of *M. bella* extracts. In particular, antidiabetic [4] and antimutagenic [2] activities were correlated with the presence of flavonoids, while antimicrobial [3] activity has been associated with the presence of hydrolyzable tannins. The existence of three distinct chemotypes revealed in this study could play a role in the pharmacological effects of the extract. Such results thus need to be considered for further quality control and bioactivity studies of *M. bella* extracts.

From a methodological viewpoint, the integration of statistical data from MVDA with the generated MN enabled clusters of compounds sharing both statistically significant level variations and structural relationships to be highlighted. Furthermore, the MN annotated through ISDB-DNP enabled the putative dereplication of secondary metabolites of *M. bella* and the additional putative identification of several compounds not previously reported for this plant [6]. This combined approach made it possible to effectively characterize three chemotypes of *M. bella* and identify their markers. Our results validated the interest in using similar approaches to assess in a comprehensive way the complex metabolic responses of plant phenotypes to genetic and environmental changes.

## 4. Materials and Methods

### 4.1. Plant Material and Sampling

Leaf samples of 271 specimens of *Myrcia bella* Cambess were collected in situ from seven different localities in Brazil, in the dry and rainy seasons of 2013, 2014, and 2015 (Table 1). The leaf samples were immediately stored after sampling in a hermetically sealed container with silica gel until they were processed in the laboratory. A voucher specimen for each population sampled was deposited at the herbarium (UNBA) of the University of São Paulo State “Júlio de Mesquita Filho”; UNESP, Brazil. A voucher specimen was also deposited at the Herbarium HUFSJ of Federal University of São João Del Rei, Minas Gerais, Brazil, under code number HUFSJ 4731, for confirmation by a *Myrcia* taxonomy specialist. The access and shipment of component of genetic heritage, as issued by the National Council for Scientific and Technological Development (Conselho Nacional de Desenvolvimento Científico e Tecnológico; CNPq), was performed under authorization No. 010468/2014-51 of Genetic Heritage Management Council (Conselho de Gestão do Patrimônio Genético—CGEN).

### 4.2. Soil Sampling and Meteorological Data

The soil collection was performed in each geographical location of plant harvesting following the protocol for composite soil samples recommended by the Soil and Environmental Resources Department of the Faculty of Agronomic Sciences (Departamento de Solos e Recursos Ambientais, UNESP-FCA). The samples were collected randomly at depths of 10–20 cm (ten replicates of each location) and a 500 g aliquot was separated, labeled, and sent for macro- and micronutrient analysis. Air-dried soil samples were analyzed for total organic carbon (OM), phosphorus (P), exchangeable Al, basic cations (K, Ca, Mg), and potential acidity (H+Al3^+^); cation exchange capacity (CEC) was determined based on the sum of K, Ca, and Mg; base saturation (V%) was calculated as a percentage of CEC; sum of bases (SB) represents Ca + Mg + K. Soil pH was determined in CaCl_2_ (0.01M) solution. The meteorological data of accumulated rainfall (mm), temperature (°C), radiation (KJ/m^2^), and humidity (%) from all respective areas of study were obtained from the available online meteorological database of the National Institute of Meteorology (Instituto Nacional de Meteorologia; INMET) between January 2013 and March 2015.

### 4.3. Sample Preparation

The collected leaf samples were dried at 45 °C until complete dryness and then ground in a knife mill grinder. An aliquot of 100 mg of the dried and powdered material was extracted with 5 mL of MeOH-H_2_O (85:15% v/v) using a ball mill (ball diameter 2 cm, frequency 30 Hz, time 5 min) (Retsch MM200) (Retsch, Haan, Germany). The extracts were purified by SPE (Finiesterre C_18_, 100 mg/1 mL) (Teknokroma Analitica, Barcelona, Spain). After cartridge conditioning (1 mL MeOH-H_2_O (85:15% v/v)), 1 mL of extract was loaded and washed to remove chlorophyll and other lipophilic pigments. Finally, an aliquot of 50 μL was diluted in 250 μL of MeOH-H_2_O (85:15% v/v).

### 4.4. UHPLC-ToF-HRMS Analysis

The chromatographic analysis was performed on a Waters Acquity ultra-performance liquid chromatography (UPLC) system coupled with a Micromass-LCT premier time-of-flight (ToF) mass spectrometer (Waters). The separations were performed using an Acquity UPLC BEH C_18_ column, 130 Å, 1.7 µm, 2.1 mm × 150 mm maintained at 60 °C. The mobile phase consisted of 0.1% formic acid in water (solvent A) and acetonitrile (solvent B) at a flow rate of 0.75 mL/min; the gradient elution was as follows: 5% B to 50% B in A in 4 min; 50–95% B in A in 3 min; 95% B in 1 min; 5 % B over 2 min. The total running time was 10 min. Injection volume was 2 µL. Data were collected by chromatographic software MassLynx 4.1TM (Waters). The electrospray ionization (ESI) conditions were set as follows: capillary voltage 2.8 kV, cone voltage 40 V, MCP detector voltage 2650 V, source temperature 120 °C. N_2_ was used as desolvation gas. The desolvation temperature was set to 250 °C at a flow rate of 600 L/h. The detection was collected between 100 and 1000 *m/z*, scanning every 0.25 s using centroid mode. A pool of all extracts (*n* = 271) was used to make quality control (samples) for instrument conditioning and stability evaluation. Experimental samples were run in a randomized order with QC injections after every fifteen experimental samples. The metabolite fingerprinting was performed in negative-ion mode to highlight phenolic compounds that are known to be present in *Myrcia bella* [5].

### 4.5. UHPLC-HRMS2 Analysis

The QC samples and reference compounds were analyzed on a Waters Acquity UPLC IClass system interfaced to a Q-Exactive Focus mass spectrometer (Thermo Scientific, Bremen, Germany), using a heated electrospray ionization (HESI-II) source. The separations were performed using an Acquity UPLC BEH C_18_ column, 130 Å, 1.7 µm, 2.1 mm × 250 mm at 60 °C. The mobile phase was 0.1% formic acid in water (solvent A) and acetonitrile (solvent B) at a flow rate of 0.75 mL/min; the gradient elution was as follows: 5% B to 100% B in A in 5 min. Injection volume was 2 µL. The optimized HESI-II parameters were set as follows: source voltage, 3.5 kV; sheath gas flow rate (N_2_), 48 units; auxiliary gas flow rate, 11 units; spare gas flow rate, 2.0 units; capillary temperature, 300 °C, S-Lens RF Level, 55. The mass analyzer was calibrated using a mixture of caffeine, methionine–arginine–phenylalanine–alanine–acetate (MRFA), sodium dodecyl sulfate, sodium taurocholate, and Ultramark 1621 in an acetonitrile/methanol/water solution containing 1% formic acid by direct injection. The data-dependent MS/MS events were performed on the three most intense ions detected in full scan MS (Top3 experiment). The MS/MS isolation window width was 2 Da, and the normalized collision energy (NCE) was set to 20/35/50 units. In data-dependent MS/MS experiments, full scans were acquired at a resolution of 35,000 fwhm (at *m/z* 200) and MS/MS scans at 17,500 fwhm, both with a maximum injection time of 50 ms. After being acquired in an MS/MS scan, parent ions were placed in a dynamic exclusion list for 3.0 s.

### 4.6. UHPLC-HRMS2 Data Processing

The UHPLC-HRMS2 raw data were converted to mzXML using the MsConverter (ProteoWizard) software and processed using MZmine 2.10 for peak detection, peak filtering, chromatogram construction, chromatogram deconvolution, isotopic peak grouping, chromatogram alignment, and gap filling. The following parameters were used for data processing: noise level at 1 × 106 for MS1 and 0 for MS2. The ADAP chromatogram builder was selected with the following parameters: minimum group size in number of scans of 5, minimum height of 1 × 10^6^, and *m/z* tolerance of 0.001 Da (or 10 ppm); chromatogram deconvolution was set as follows: wavelets (ADAP) was used as the algorithm for peak recognition, *m/z* and RT range for MS2 scan pairing were 0.3 Da and 0.1 min, S/N threshold was 50, minimum feature height was 5 × 10^5^, coefficient/area threshold was 90, peak duration range was 0.02–1.5 min, and the RT wavelet range was 0.02–0.05. Chromatograms were then deisotoped by isotopic peaks a grouper algorithm with a *m/z* tolerance of 0.001 Da and an RT tolerance of 0.05 min. Peak alignment was carried out using a join aligner, with *m/z* tolerance set at 0.001 Da, absolute RT tolerance at 0.05 min, and weight for *m/z* and RT at 30. The missing peaklist after alignment was filled by gap filling of the same RT and *m/z* range gap-filler module with a *m/z* tolerance of 0.001 Da. After gap filling, all peaklists were done with identification of adduct search, complex search, and molecular formula prediction. This resulted in a peaklist of 1296 features with associated data-dependent MS2 spectra. This resulting peaklist was exported as input for MN generation.

### 4.7. Molecular Network Analysis and Computational Annotation

The molecular network (MN) was generated using the feature-based molecular networking workflow (FBMN) of the Global Natural Products Social Molecular Networking (GNPS) (http://gnps.ucsd.edu/). A unique MN was then created where edges were filtered to have a cosine score above 0.7 and more than six matched peaks. Further edges between two nodes were kept in the network if and only if each of the nodes appeared in each other’s respective top 10 most similar nodes. The spectra in the network were then searched against GNPS spectral libraries. All matches kept between network spectra and library spectra were required to have a score above 0.7 and at least six matched peaks. The spectra in the molecular network were then searched automatically against the ISDB-DNP (In Silico Data Base-Dictionary of Natural Products) spectral library using the workflow described in Allard et al. [19]. The parameters used to process the spectral dataset analysis were as follows: parent mass tolerance 0.05 Da, minimum cosine score 0.1, returning top 50 candidates. Scripts for the ISDB-DNP metabolite annotation process are available at the following address: https://github.com/oolonek/ISDB.

### 4.8. Taxonomically Informed Scoring

A taxonomically informed scoring script was used to re-rank the annotations returned by ISDB-DNP [30]. The top 50 candidates were re-ranked based on taxonomic data associated with the queried spectra (Myrtaceae > *Myrcia* > *Myrcia bella*) and the biological source of the candidate structures. Scripts for the taxonomically informed metabolite annotation are available at the following address: https://github.com/oolonek/taxo_scorer. The structure of the compound returned by the taxonomically informed scoring annotation at rank 1 was visualized in the MN using the ChemViz 1.3 plugin (freely available at http://www.cgl.ucsf.edu/cytoscape/chemViz/) directly within Cytoscape 3.7.2.

### 4.9. DNA Extraction and Amplified Fragment Length Polymorphism (AFLP) Analysis

Amplified fragment length polymorphism analysis was performed in a representative subset of 40 samples from six populations. These samples were selected based on sample availability and on the quality of the DNA material extracted, seen as a unique band close to the well (indentations) at one end of a gel electrophoresis in which each extract was deposited. Electrophoresis was also used to estimate DNA quantities, for dilutions purposes. Only samples from the Bonito (BT) location, from Mato Grosso do Sul state, were unavailable to perform this analysis.

An aliquot of 1 g of the leaves were harvested and stored in hermetic bags with silica gel for the DNA extraction. The samples were stored in a freezer at -80°C. The DNA was extracted from silica gel dried leaf samples (30–50 mg) using the CTAB method, as described by Ferreira and Grattapaglia [70].

The AFLP was carried out according to the AFLPTM Plant Mapping protocol of Applied Biosystems, with modification [71]. Total DNA cleavage reaction was performed using 2 μL of buffer T4 DNA ligase, 0.20 μL of MseI (50 U/mL), 0.50 μL of EcoRI (10 U/mL) for 2 h at 37 °C, 15 min at 70 °C, 30 min at 20 °C. For binding reaction 5.5 μL of cleavage reaction product were added to 2 μL of buffer T4 DNA ligase 5× (Invitrogen), 1 μL of 0.5 M NaCl, 0.5 μL of BSA, 1 μL of each of the adapters for MseI and EcoRI (Applied Biosystems), and 1 μL of the enzyme T4 DNA ligase (Invitrogen). The mixture was incubated overnight at between 15–20 °C. This amplification was made using 2 μL of a diluted aliquot (1:10) of binding reaction product, 7.5 μL of core soln. mix (Applied Biosystems), and 0.5 μL of primers. The amplification cycles started with 2 min at 72 °C, followed by 20 cycles, of 20 s at 94 °C, 30 s at 56 °C, and 2 min at 72 °C, and ended at 60 °C for 30 min. For selective amplification, 1.5 μL of a diluted aliquot (1:10) of pre-selective reaction product was added to 7.5 μL of core solution mix (Applied Biosystems) and 0.5 μL of each MseI and EcoRI primers combined. The selective amplification cycles started with 2 min at 94 °C, followed 10 cycles of 20 s at 94 °C, 30 s at 66 °C with reduction of 1 °C/s, and 2 min at 72°C, followed 20 cycles of 20 s at 94 °C, 30 s at 56 °C, and 2 min at 72 °C, and ended at 60 °C for 30 min.

The analysis of the generated fragments was done using the Applied Biosystems 3730 DNA Analyser (Applied Biosystems, Foster city, USA), following the AFLP Plant Mapping Protocol [71]. For the analysis of the fragments, 1.5 μL of the amplified product, 11 μL of formamide, and 0.5 μL of standard fragment sizes (ROX) were mixed and heated at 95 °C for 5 min. The fragments detected were analyzed by size of base pairs (bp) using Genescan 500 ROX Standard.

### 4.10. Genetic Data

Several primer combinations were tested for *M. bella* samples, and those with higher number of fragments were selected. The final AFLP analysis was performed using four primer combinations, Eco-ACC/MseI-CAT (291 Allele); Eco-ACG/MseI-CAG (196 Allele), Eco-ACC/MseI-CAA (316 Allele), and Eco-ACG/MseI-CTG (225 Allele). The raw data were analyzed using the ABI Prism Genescan analysis software [72]. Fragments of 50–500 bp were scored as present (1) or absent (0) using ABI Prism Genotyper 2.5 Software (Applied Biosystems, Foster city, USA) [73], and then submitted to multivariate analysis. Percentage of polymorphic bands (P), total genotypic diversity (He) [32,33], were calculated using GenAlEx 6.3.

### 4.11. Multivariate Data Analysis

The UPLC-ToF-HRMS data (retention time and MS signal intensities) were processed using MZmine 2.10 (manufacturer, city, country) for peak detection, peak filtering, chromatogram construction, chromatogram deconvolution, isotopic peak grouping, chromatogram alignment, and gap filling. The following parameters were used for data processing: noise level at 106, mass tolerance 0.02 (Da), intensity threshold 250 (counts), and mass window 0.05. A peak list of 271 × 704 matrix was obtained.

The obtained peaklist was pre-treated based on the repeatability of signal intensity from the QC samples using the coefficient of variation (CV). Ions with CV < 30% were filtered. This procedure generated a final peaklist with 302 (42.9%) ions. This final dataset of a 302 × 271 matrix was then imported to SIMCA-P 14.1 (Umetrics^®^) (Sartorius, Umeå, Sweden).

Principal component analysis (PCA) was generated with the final dataset as X input with Pareto variance scaling. A two-way orthogonal partial least squares (O2PLS) analysis in multi-block (X and Y) modeling was performed [27]. For the O2PLS analysis, the X input was complemented with the meteorological and soil data as Y input. The features with discriminant potential from the O2PLS model were selected based on their variable of importance in the projection (VIP) values. A threshold value (VIP > 1.0) was applied.

The AFLP data were treated using the ABI Prism Genotyper 2.5 Software (Applied Biosystems, Foster city, USA), and a matrix containing 1028 alleles scored as present (1) or absent (0) was submitted to PCA and HCA analysis using unit variance scaling. Hierarchical clustering analysis was performed based on Euclidean distance.

The soil dataset matrix (17 × 7) containing mineral and nutrient compositions of each of the seven areas was submitted to PCA and PCA-biplot analysis with unit variance scaling.

The correlation analysis and correlation *p*-values were conducted using the ggplot2 package in the R Statistical Software version 4.0 [74].

### 4.12. Data Availability

The full HRMS2 dataset is uploaded and accessible on the GNPS server as massive data in ftp://massive.ucsd.edu/MSV000085139/. The metabolomics raw data (UHPLC-ToF-HRMS) are deposited in MetaboLights (http://www.ebi.ac.uk/metabolights) under the identifier code MTBLS1728.

## Figures and Tables

**Table 1:** Sampling plan of *Myrcia bella* specimens compared in this study. City and exact coordinates for each sampled area of Cerrado are presented. Samples were collected twice per season, the months and years of each harvest period are given. Only samples from Parque Nacional das Emas-Goiás were collected once in each season. The total number of samples collected in each area in both seasons is given. A representative specimen voucher for each population was deposited at the UNBA herbarium.

City (State)	Code	Coordinates	Voucher Code	Samples	Season	
					Dry	Rainy
Bonito (MS)	BT	S 21°07′15″, W 56°28′55″	6031	32	June/2013 July/2014	March/2013 April/2015
Campo Grande (MS)	CG	S 20°30′29.3″, W 54°361′59.3″	6033	25	June/2013 July/2014	March/2013 May/2015
Jardim Botânico de Bauru (SP)	JBB	S 22°20′30″, W 49° 00′30″	5508	55	August/2013 July/2014	May/2013 May/2015
Parque Nacional das Emas (GO)	PNE	S 18°07′17″, W 52°54′30″	6028	29	June/2014	March/2015
Pratânia (SP)	PT	S 22°48′28″, W 48°39′57″	6029	50	September/2013 July/2014	May/2013 March/2015
Selvíria (MS)	S	S 20°64.4′73.6″, W 51°76.4′92.4″	6032	33	June/2013 July/2014	March/2013 April/2015
Três Lagoas (MS)	TL	S 20°46′39.5″, W 51°40′25.5′’	6030	47	June/2013 July/2014	March/2013 April/2015
				Total: 271		

List of abbreviations: MS = Mato Grosso do Sul; SP = São Paulo; GO = Goiás.

**Table 2:** Parameters of intrapopulation genetic diversity of *Myrcia bella* based on AFLP markers.

Locality	Na	P (%)	He
CG	5	20.72	0.059
JBB	9	50.78	0.124
PNE	10	54.77	0.135
PT	8	45.91	0.116
S	3	11.58	0.048
TL	5	38.13	0.107

List of abbreviations: Na = sample size, P = percentage of polymorphic loci, He = gene diversity of Nei. CG = Campo Grande; S = Selvíria; TL = Três Lagoas; PNE = Parque Nacional das Emas; PT = Pratânia; JBB = Jardim Botânico de Bauru.

**Table 3:** Genetic distance of *Myrcia bella* populations from different regions of Cerrado.

	CG	JBB	PNE	PT	S	TL
**CG**	0.000					
**JBB**	0.040	0.000				
**PNE**	0.033	0.022	0.000			
**PT**	0.021	0.026	0.028	0.000		
**S**	0.086	0.099	0.082	0.089	0.000	
**TL**	0.022	0.035	0.028	0.025	0.078	0.000

List of abbreviations: CG = Campo Grande; S = Selvíria; TL = Três Lagoas; PNE = Parque Nacional das Emas; PT = Pratânia; JBB = Jardim Botânico de Bauru.

## Supplementary Materials

The following are available online, Figure S1: Meteorological data of the seven areas of harvest recorded during the 24 month period of this study, Table S1: Micro- and macronutrient levels in the soil of the harvested areas, Table S2: Mineral composition of the soil of the harvested areas, Figure S2: Chromatograms of *Myrcia bella* quality control samples (QC) used to evaluate the UHPLC-ToF-MS instrument performance during the metabolomics experiment over 72 h of analysis, Figure S3: PCA score plot of UHPLC-ToF-HRMS data of all samples. (a) Data non-log-transformed, (b) Data log-transformed, Figure S4: HCA dendrogram for UHPLC-ToF-HRMS data obtained from *Myrcia bella* populations from different locations, Figure S5: Multivariate data analysis of soil data of all areas studied. (a) PCA score scatter plot based on soil nutrients and mineral data. (b) PCA-biplot exhibiting the correlation of the soil mineral composition as well as macro- and micronutrients within the harvested areas, Figure S6: Separate PCA score plots of each of *Myrcia bella* population colored by dry (green) and rainy (blue) seasons from all areas of study, Figure S7: Comparison of the obtained MS2 spectra from *Myrcia bella* extract with authentic standard spectra, Figure S8: *Statistically-informed molecular networking* generated by integrating metabolomics MVDA to the MN, Table S4: Identification of compounds in *Myrcia bella* leaf extracts by UHPLC-HRMS2 analysis in negative mode, Figure S9: Correlation matrix of features with meteorological and soil data, Figure S10: Variable plot line from the MVDA of the selected compounds and their relative intensities for each locality, Figure S11: 1,5% agarose gel electrophoresis of DNA obtained from the leaves of specimens of *Myrcia bella* collected in different regions of the Cerrado.
