# Supplementary material for: Metabolomics of Myrcia bella Populations in Brazilian Savanna Reveals Strong Influence of Environmental Factors on Its Specialized Metabolism
Source: Molecules. 2020 Jun 26;25(12):2954. doi: 10.3390/molecules25122954 (PMC7356273; doi:10.3390/molecules25122954)
Supplement: Supplementary file 1 [file molecules-25-02954-s001.pdf]

molecules

Supplementary materials

Metabolomics of *Myrcia bella* Populations in Brazilian Savanna Reveals Strong Influence of Environmental Factors on Its Specialized Metabolism

Luiz Leonardo Saldanha [1,2,*], Pierre-Marie Allard [2], Adlin Afzan [2], Fernanda Pereira de Souza Rosa de Melo [1], Laurence Marcourt [2], Emerson Ferreira Queiroz [2], Wagner Vilegas [3], Cláudia Maria Furlan [4], Anne Lígia Dokkedal [1] and Jean-Luc Wolfender [2,*]

[1] Faculty of Sciences, São Paulo State University (UNESP), CEP 17033-360, Bauru, São Paulo, Brazil
[2] School of Pharmaceutical Sciences, Institute of Pharmaceutical Sciences of Western Switzerland, University of Geneva (IPSWS), CH-1211 Geneva 4, Switzerland
[3] Institute of Biosciences, São Paulo State University (UNESP), CEP 11330-900, São Vicente, São Paulo, Brazil
[4] Institute of Biosciences, University of São Paulo, CEP 05508-090, São Paulo, São Paulo, Brazil
* Correspondence: lluizsaldanha@gmail.com (L.L.S.); jean-luc.wolfender@unige.ch (J.-L.W.); Tel.: +55-19-3526-4194 (L.L.S.); +41-22-379-3385 (J.-L.W.)

1. Meteorological Data

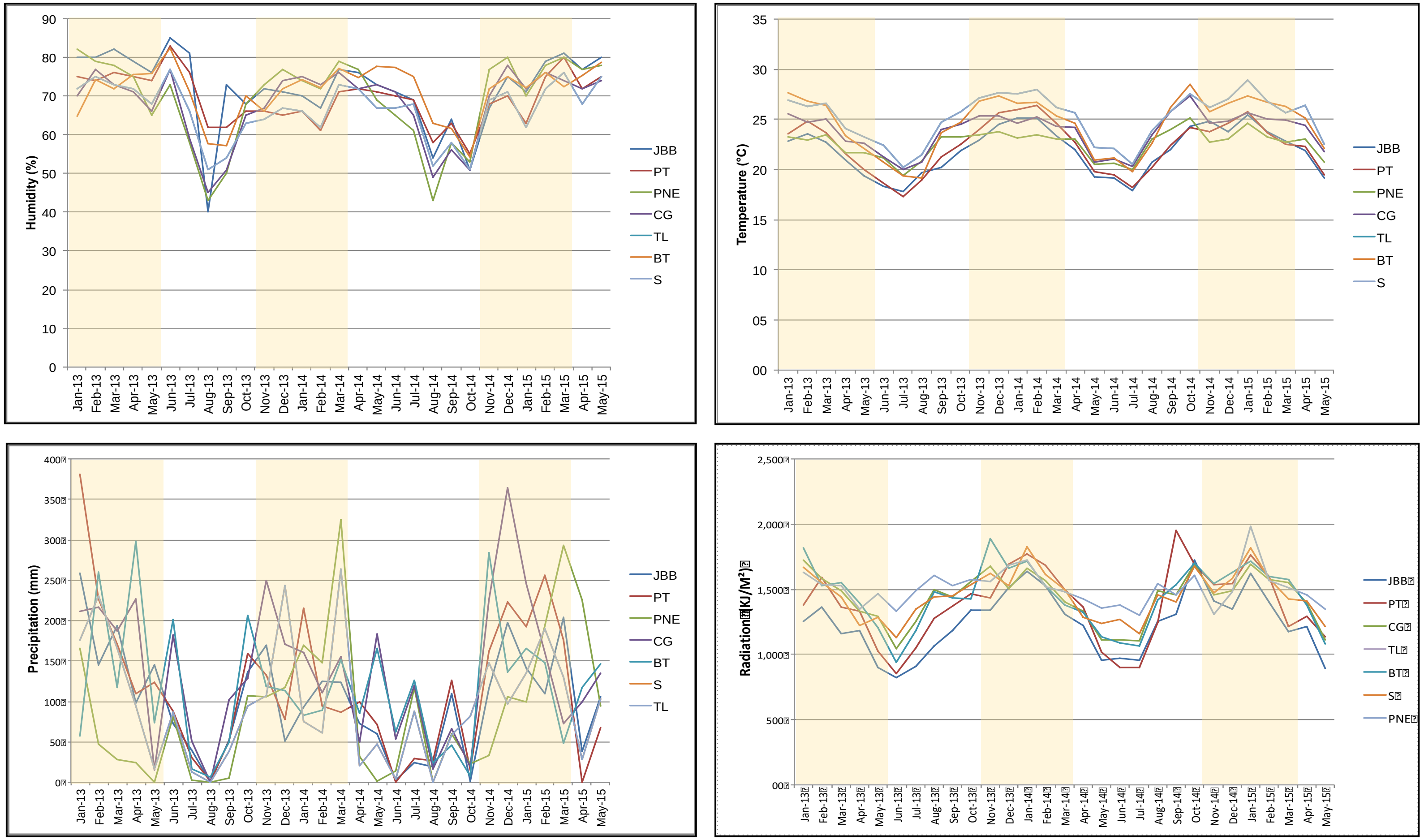

Figure S1. Meteorological data of the seven areas of harvest recorded during the 24 month period of this study. Precipitation is given by total mean precipitation by month. Gray area indicates rainy season and withe area dry season. List of abbreviations: JBB = Jardim Botânico de Bauru; PT = Pratânia; PNE = Parque Nacional das Emas; CG = Campo Grande; BT = Bonito; S = Selvíria; TL = Três Lagoas.

2. Soil Properties Data

Table S1. Micro- and macronutrient levels in the soil of the harvested areas.

Harvest Area/State	Al	K	Ca	Mg	SB	B	Cu	Fe	Mn	Zn
CG/MS	16	0,7	10	2	13	1,11	2,3	24	38,0	0,2
BT/MS	1	2,7	23	11	37	0,33	6,2	33	4,4	1,4
S/MS	7	0,6	10	3	14	0,92	0,3	8	1,5	0,1
TL/MS	4	0,5	13	4	17	0,59	0,2	24	0,9	0,1
PNE/GO	8	0,6	11	3	15	0,43	0,2	61	0,7	0,3
PT/SP	7	1,3	10	3	15	0,84	0,3	54	4,0	1,1
JBB/SP	9	0,6	11	3	15	0,87	0,5	38	2,0	0,1

P, Fe, Zn, Mn, B and S given as mg/dm^3; K, Ca and Mg given as $mmol_c/dm^3$. List of abbreviations: BT = Bonito; CG = Campo Grande; S = Selvíria; TL = Três Lagoas; PNE = Parque Nacional das Emas; PT = Pratânia; JBB = Jardim Botânico de Bauru. GO = Goiás; SP = São Paulo; MS = Mato Grosso do Sul. Fe = Soil iron; Al = Soil aluminum; Mn = Soil manganese; K = Soil potassium; Cu = Soil copper; Mg = Soil magnesium; Ca = Soil calcium; Zn = Soil zinc; SB = Soil sum of basis; pH = Soil pH; V = Soil bases saturation; Temp = air temperature; Temp max = air temperature maximum. BT = Bonito; CG = Campo Grande; S = Selvíria; TL = Três Lagoas; PNE = Parque Nacional das Emas; PT = Pratânia; JBB = Jardim Botânico de Bauru. GO = Goiás; SP = São Paulo; MS = Mato Grosso do Sul.

Table S2. Mineral composition of the soil of the harvested areas.

Harvest Area/State	pH ($CaCl_2$)	OM	P	H + Al	SB	CEC	V
CG/MS	4,0	23	15	83	13	96	13
BT/MS	5,0	59	8	51	37	88	42
S/MS	4,7	13	5	27	14	41	34
TL/MS	4,7	6	3	18	17	35	48
PNE/GO	4,2	26	3	55	15	70	22
PT/SP	4,3	13	4	33	15	47	31
JBB/SP	4,1	12	3	41	15	57	27

Organic matter (OM) given as g/dm^3; Cation exchange capacity (CEC) given as % and potential acidity (H+Al) as $mmol_c/dm^3$; Sum of bases (SB) given as %. V = base saturation given as %. P = Soil phosphorus. List of abbreviations: BT = Bonito; CG = Campo Grande; S = Selvíria; TL = Três Lagoas; PNE = Parque Nacional das Emas; PT = Pratânia; JBB = Jardim Botânico de Bauru. GO = Goiás; SP = São Paulo; MS = Mato Grosso do Sul.

3. Chromatograms of *Myrcia Bella* Quality Control Samples (QC)

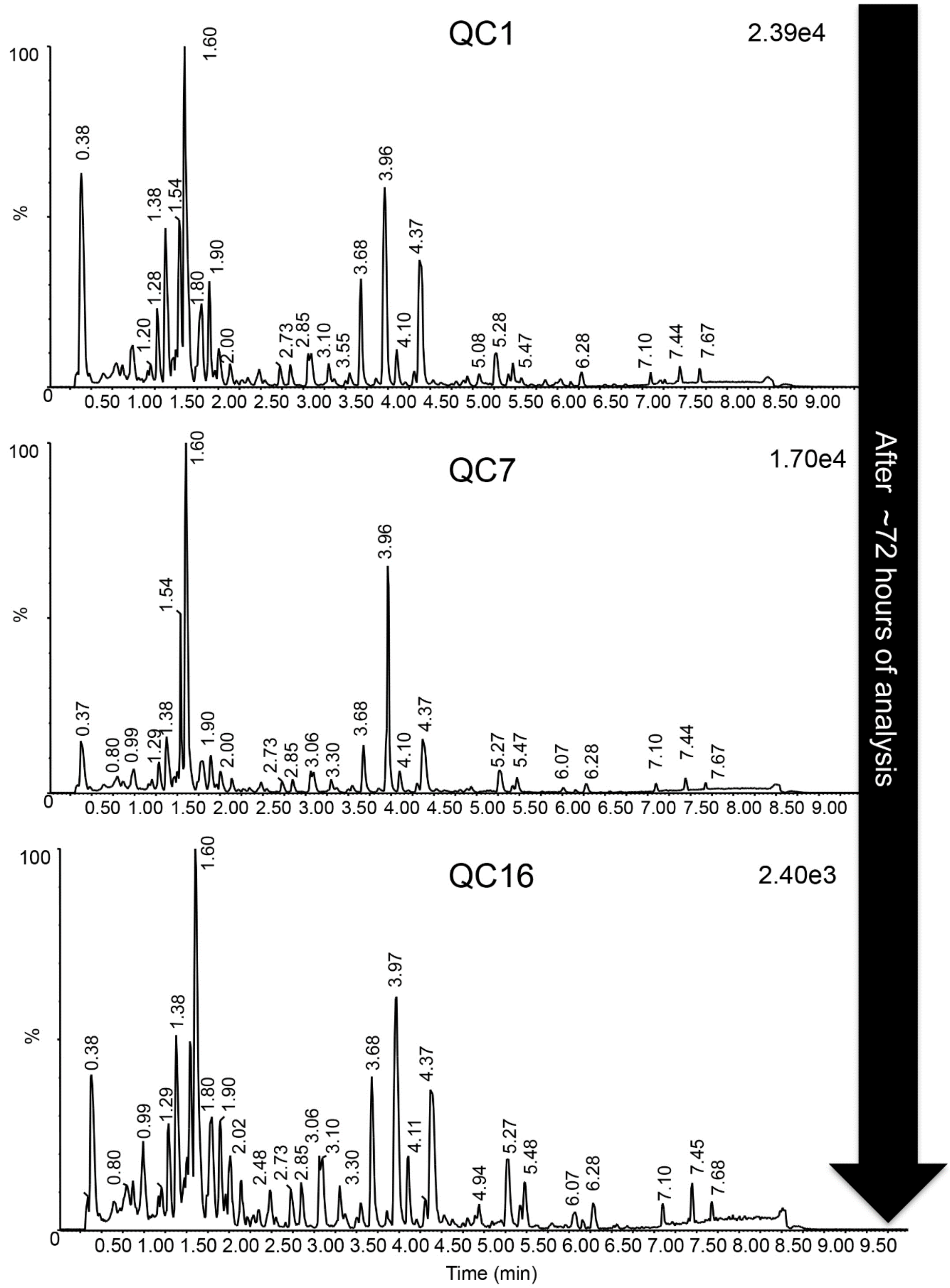

Figure S2 Chromatograms of *Myrcia bella* quality control samples (QC) used to evaluate the UHPLC-ToF-MS instrument performance during the metabolomics experiment over 72 h of analysis.

4. PCA Scores Plot of UHPLC-ToF-HRMS

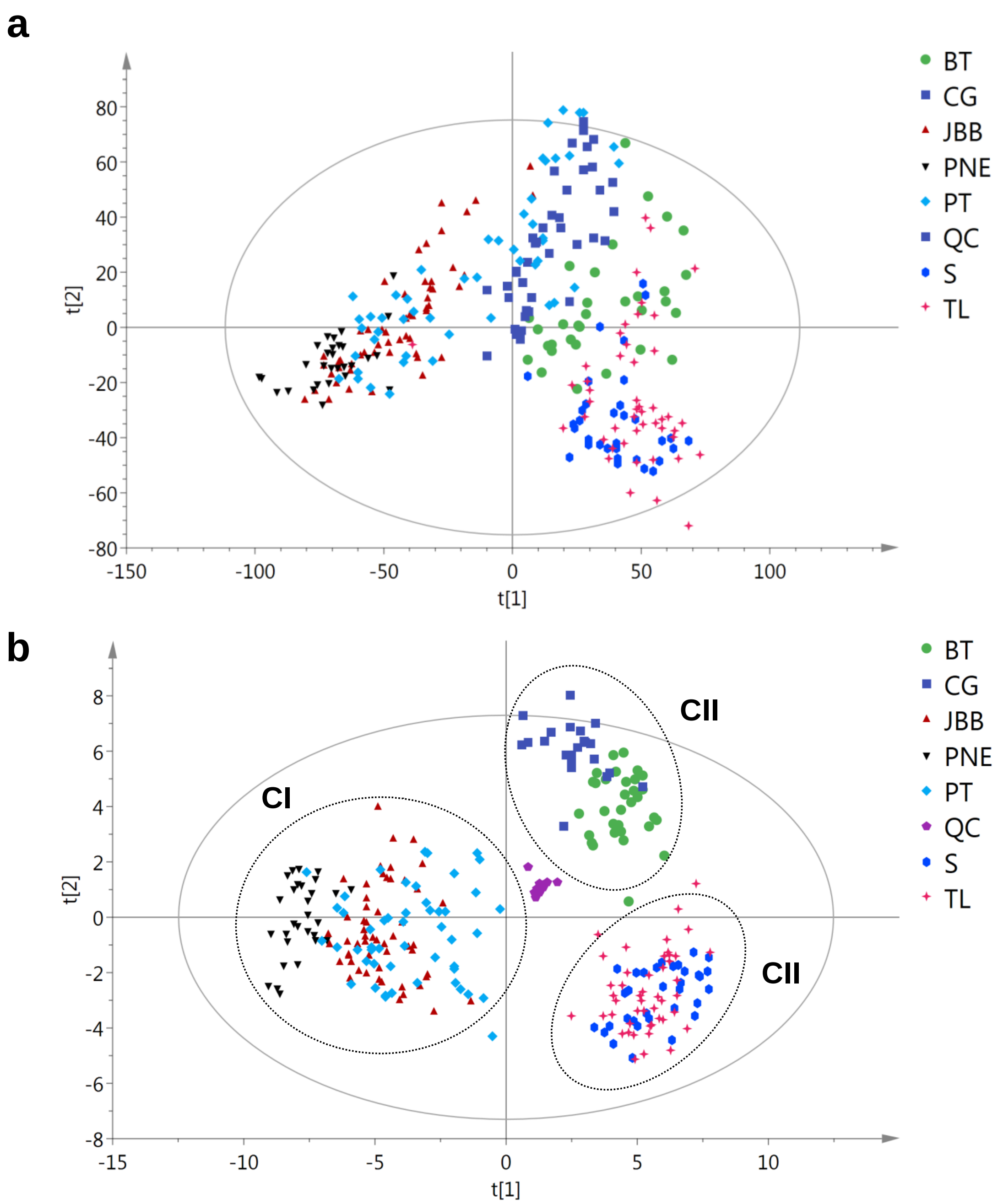

Figure S3 PCA scores plot of UHPLC-ToF-HRMS data of all samples. (**a**) Data non log transformed (**b**) Data log transformed. Dotted line boxes indicate the identified chemotypes CI, CII and CIII. List of abbreviations: BT = Bonito; CG = Campo Grande; JBB = Jardim Botânico de Bauru; PNE = Parque Nacional das Emas; PT = Pratânia; QC = quality control samples; S = Selvíria; TL = Três Lagoas.

5. HCA dendrogram for UHPLC-ToF-HRMS data

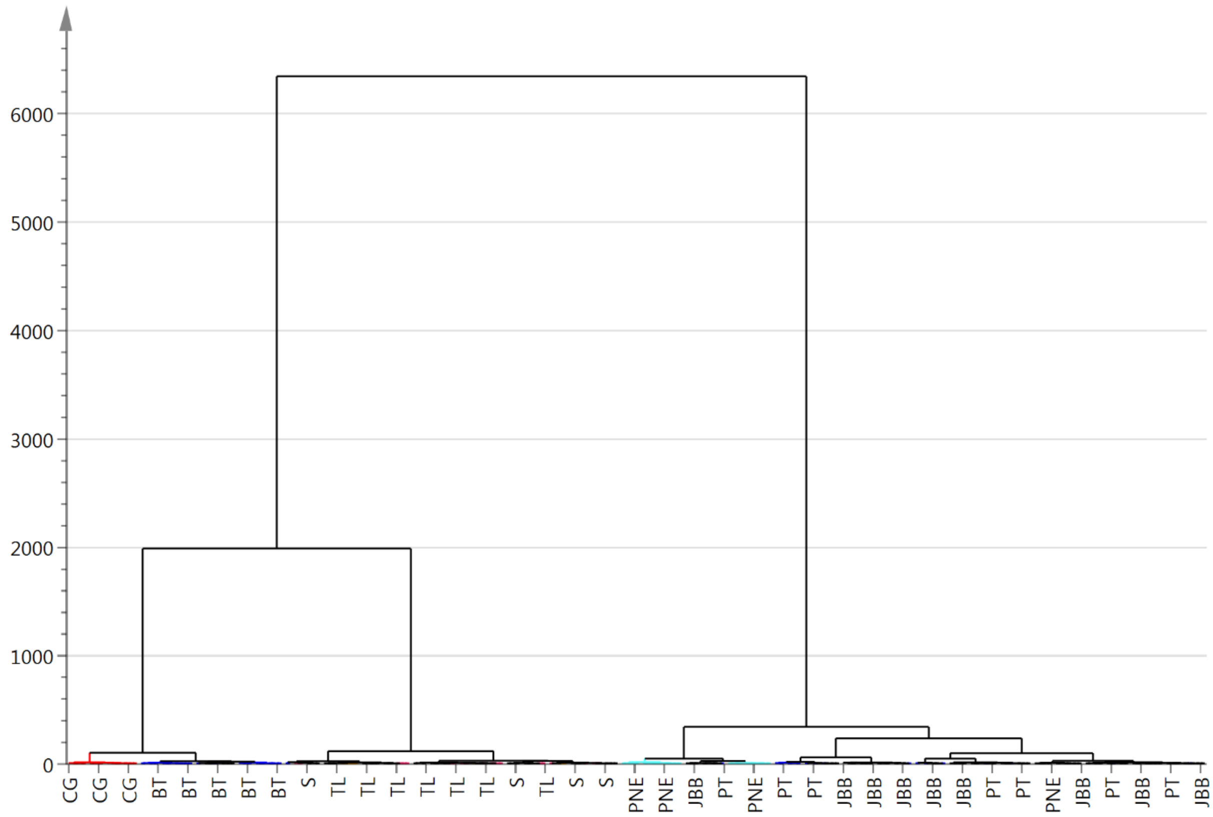

Figure S4 HCA dendrogram for UHPLC-ToF-HRMS data obtained from *Myrcia bella* populations from different localities. List of abbreviations: BT = Bonito; CG = Campo Grande; JBB = Jardim Botânico de Bauru; PNE = Parque Nacional das Emas; PT = Pratânia; S = Selvíria; TL = Três Lagoas. GO = Goiás; SP = São Paulo; MS = Mato Grosso do Sul.

6. Multivariate data analysis of soil properties

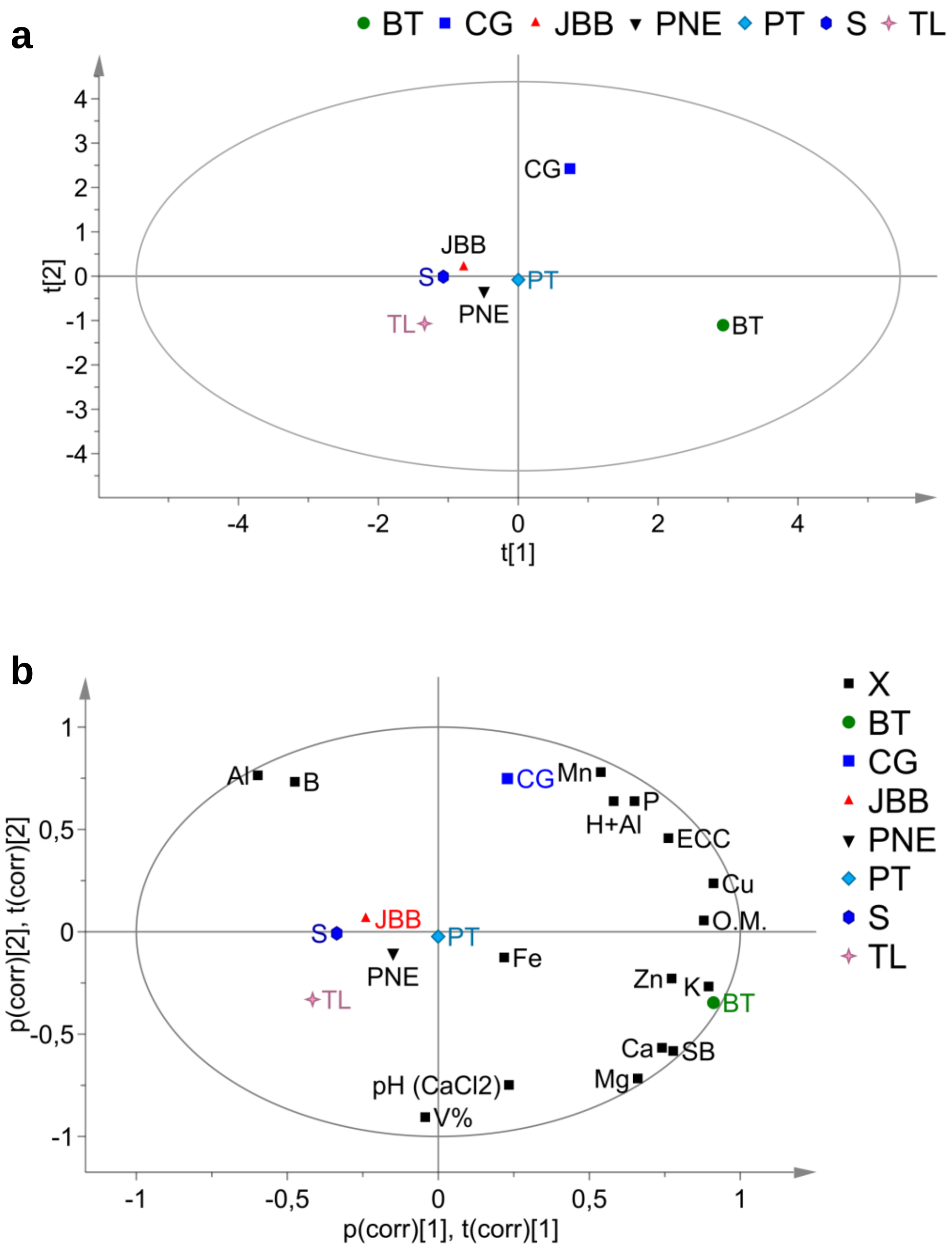

Figure S5 Multivariate data analysis of soil data of all areas studied. (**a**) PCA score scatter plot based on soil nutrients and mineral data. (**b**). PCA-biplot exhibiting the correlation of the soil mineral composition as well as macro and micronutrients within the harvested areas. List of abbreviations: Fe = Soil iron; Al = Soil aluminum; Mn = Soil manganese; K = Soil potassium; Cu = Soil copper; P = Soil phosphorus; Mg = Soil magnesium; Zn = Soil zinc; Ca = Soil calcium; SB = Soil sum of basis; pH = Soil pH; V = Soil bases saturation; ECC = cation exchange capacity. BT = Bonito; CG = Campo Grande; S = Selvíria; TL = Três Lagoas; PNE = Parque Nacional das Emas; PT = Pratânia; JBB = Jardim Botânico de Bauru. GO = Goiás; SP = São Paulo; MS = Mato Grosso do Sul.

7. Multivariate data analysis of dry and rainy seasons

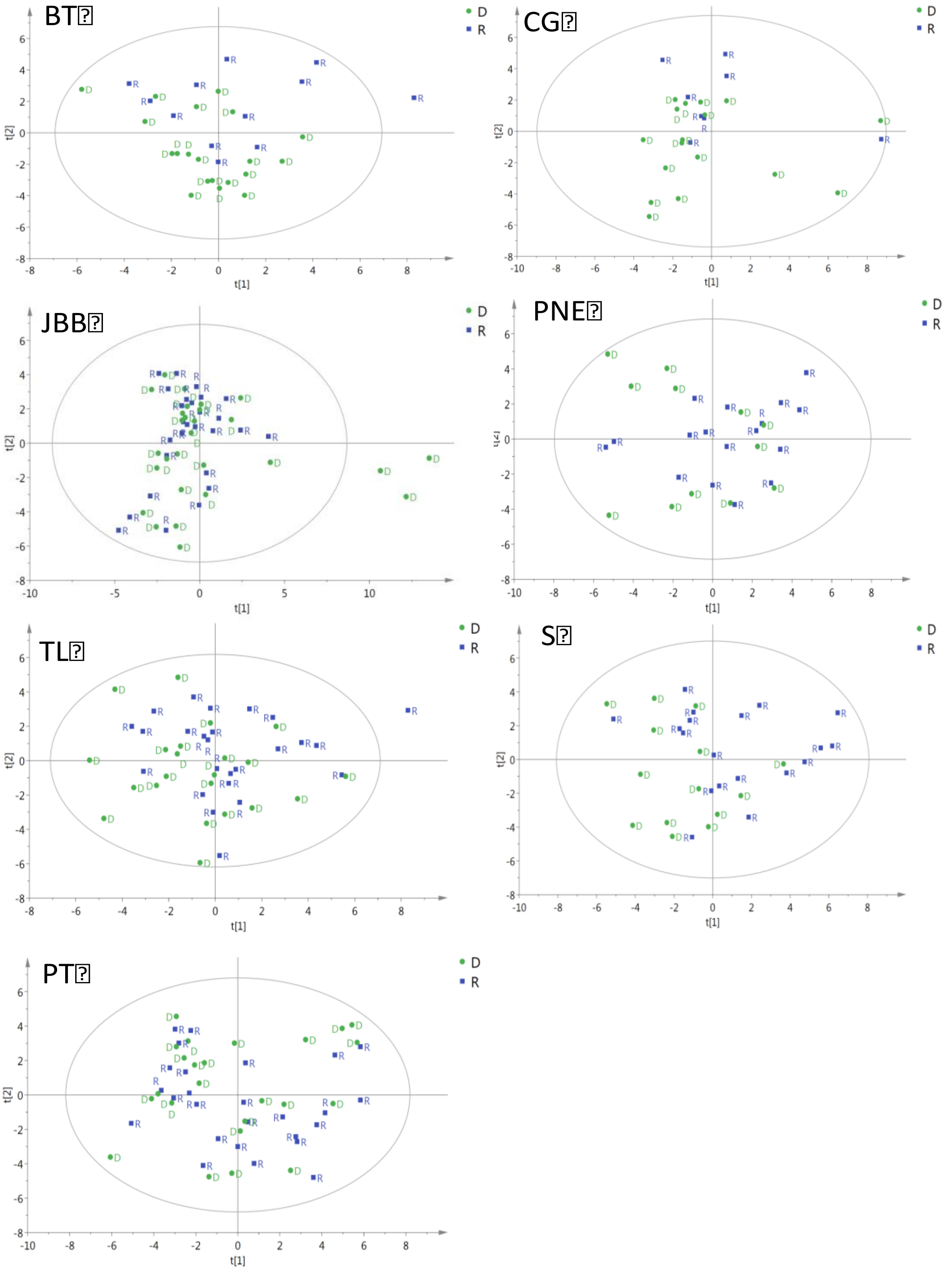

Figure S6 Separate PCA score plot of each of *Myrcia bella* populations colored by dry (green) and rainy (blue) seasons from all areas of study. List of abbreviations: BT = Bonito; CG = Campo Grande; S = Selviria; TL = Tres Lagoas; PNE = Parque Nacional das Emas; PT = Pratânia; JBB = Jardim Botânico de Bauru.

8. Comparison of the obtained spectra with authentic standards

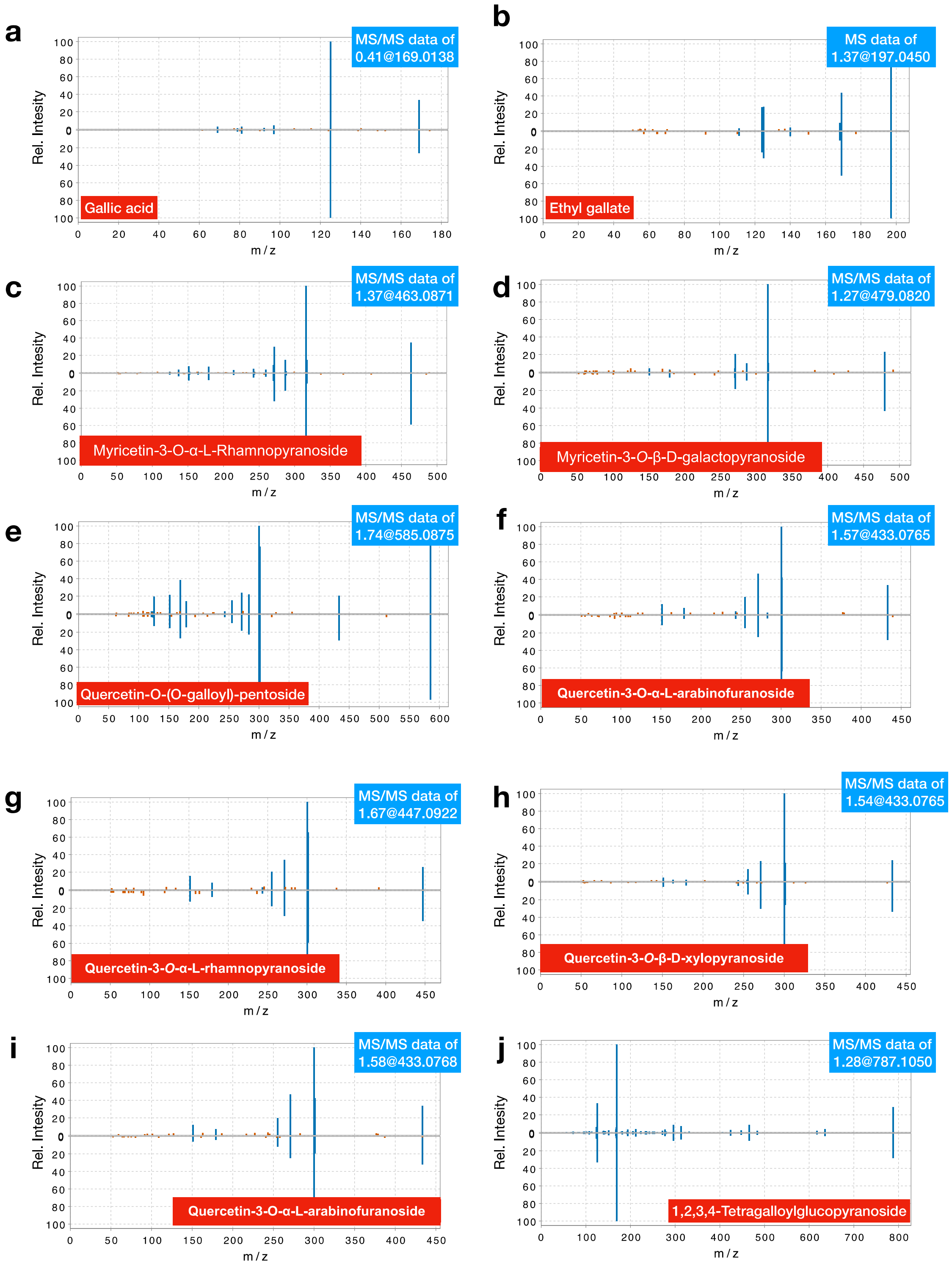

Figure S7. Comparison of the obtained MS^2 spectra from *Myrcia bella* extract with authentic standards spectra. (**a**) Spectra comparison for gallic acid. (**b**) Spectra comparison for ethyl gallate. (**c**) Spectra comparison for Myricetin-3-O-α-L-Rhamnopyranoside. (**d**) Spectra comparison for Myricetin-3-O-β-D-galactopyranoside. (**e**) Spectra comparison for Quercetin-O-(O-galloyl)-pentoside. (**f**) Spectra comparison for Quercetin-3-O-α-L-arabinofuranoside. (**g**) Spectra comparison for Quercetin-O-α-L -rhamnopyranoside. (**h**) Spectra comparison for Quercetin-3-O-β-D-xylopyranoside. (**i**) Spectra comparison for Quercetin-3-O-a-L-arabinofuranoside. (**j**) Spectra comparison for 1,2,3,4-Tetragalloylglucopyranoside.

9. Molecular Networking Analysis

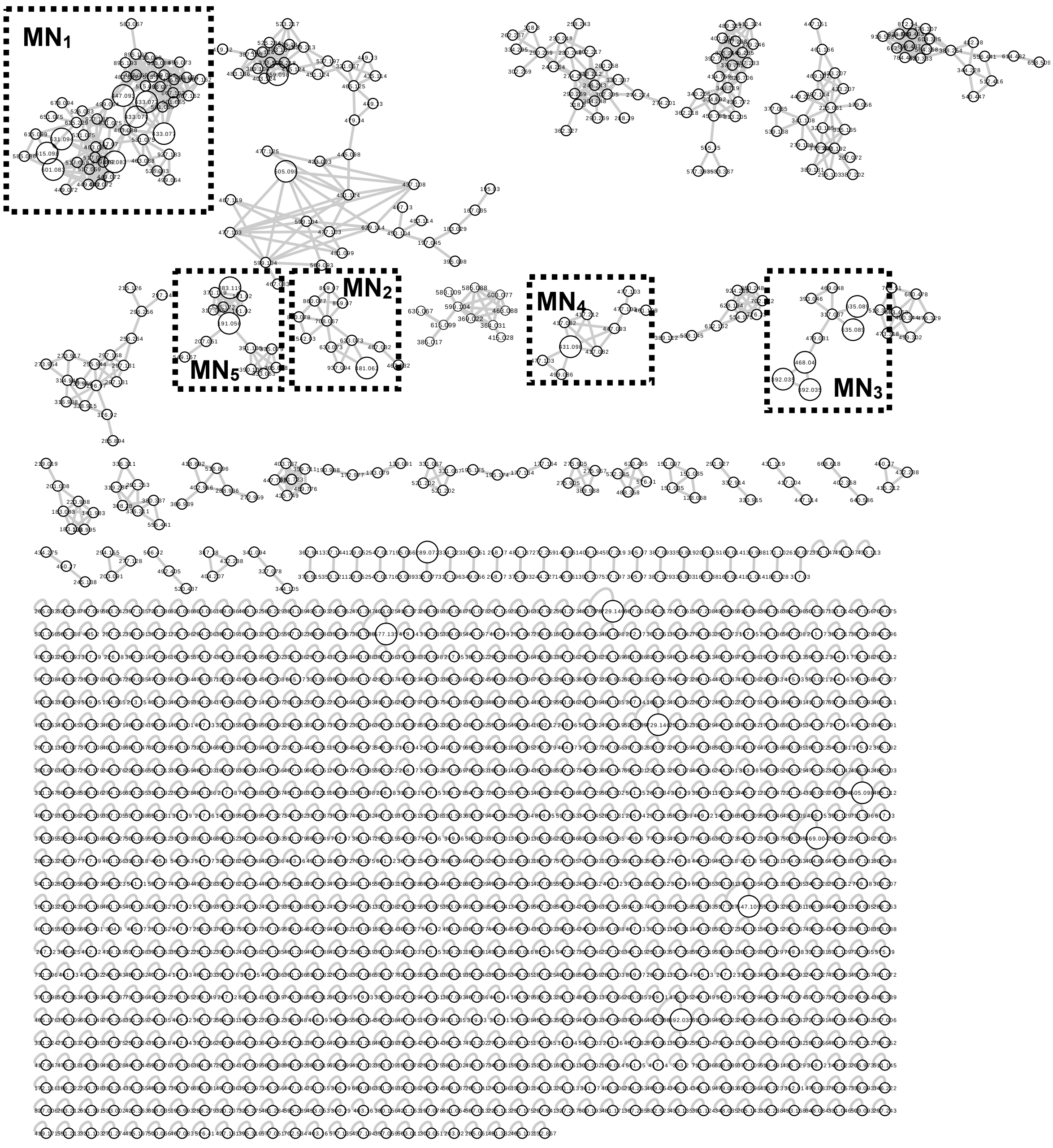

Figure S8. *Statistically-informed molecular networking* generated by integrating metabolomics MVDA to the MN. The VIP values from the O2PLS analysis were merged in the MN and can be visualized through the size of the node. Nodes with higher size indicate features with VIP values > 1. Clusters (MN_1-MN_5) were selected based on their node size and are highlighted in dotted line boxes.

10. Annotated compounds in the selected clusters from the MN

Table S4. Identification of compounds in *Myrcia bella* leaf extracts by UHPLC-HRMS2 analysis in negative mode. Compounds isolated from *M. bella* leaves were used as reference compounds to confirm the annotated compounds when available. The partial InChIKey (International Chemical Identifier) for the annotated metabolites is presented.

No.	*m/z*	Molecular formula (neutral)	Partial InChIKey	Annotation (correspondent compound in DNP or GNPS databases)	Confirmation with Authentic Standards (Co-injection)	Classification
1	191.0697	$C_{11}H_{11}O_3$	YYPABUKAWQJAHV	D(-)-quinic acid		CA
2	331.0669	$C_{13}H_{16}O_{10}$	VGVDLJNNDOFWKT	6-Galloylglucose		HT
3	633.0732	$C_{27}H_{21}O_{18}$	USKARXYQJDURNC	3,6-(HHDP)-glucose		HT
4	331.0669	$C_{13}H_{15}O_{10}$	VGVDLJNNDOFWKT	6-Galloylglucose		HT
5	633.0732	$C_{27}H_{21}O_{18}$	USKARXYQJDURNC	3,6-(HHDP)-glucose		HT
6	169.0140	$C_7H_6O_5$	BRRSNXCXLSVPFC	Gallic acid	Gallic acid	CA
7	331.0669	$C_{13}H_{15}O_{10}$,	VGVDLJNNDOFWKT	6-Galloylglucose		HT
8	633.0725	$C_{27}H_{21}O_{18}$	USKARXYQJDURNC	3,6-(HHDP)-glucose		HT
9	449.1297	$C_{18}H_{26}O_{13}$	AAJPDFHYCHTALO	3-Hydroxy-2-methyl-4H-pyran-4-oneO-[β-D-Glucopyranosyl-(1→6)-β-D-glucopyranoside]		CA
10	497.1298	$C_{22}H_{26}O_{13}$	CXAWVDXUHIJTBI	3-O-Caffeoylshikimic acid-4-O-beta-D-Glucopyranoside		CA
11	315.0721	$C_{13}H_{16}O_9$	JBLVGFKMCLXCOP	3,4-Dihydroxybenzoic acid-4-*O*-β-D-Glucopyranoside		CA
12	419.1195	$C_{17}H_{24}O_{12}$	JVCXUCHSIDSOMM	3-Hydroxy-2-methyl-4H-pyran-4-one-*O*-[β-D-Xylopyranosyl-(1→6)-β-D-glucopyranoside]		CA
13	153.0191	$C_7H_6O_4$	UIAFKZKHHVMJGS	3,4-Dihydroxybenzoic acid		CA
14	783.0677	C_{37} H_{30} O_{16}	UJWNZRSDSZZIKI	BIS-HHDP-glucose		HT
15	389.1084	$C_{16}H_{22}O_{11}$	GOZMBEYOICLJFA	1,2-Propanediol(S)-form1-O-[3,4,5-Trihydroxybenzoyl-(→6)-β-D-glucopyranoside]		CA
16	285.0614	$C_{12}H_{14}O_8$	PKCGDMKATCKBQD	3,4-Dihydroxybenzoic acid- 3-O-β-D-Xylopyranoside		CA
17	481.0619	$C_{20}H_{18}O_{14}$	GEAGRKQCZVLNAU	2,3-(*S*)-HHDP-glucose		HT
18	359.0977	$C_{15}H_{20}O_{10}$	RBSWIJOTZSNHNH	6-(3,5-Di-*O*-methylgalloyl)- β-D-glucopyranoside		CA
19	635.0881	$C_{27}H_{24}O_{18}$	MACFXELYCBWKGT	1,2,3-Trigalloyl-O-β-D-glucopyranoside		HT
20	359.0980	$C_{15}H_{20}O_{10}$	RBSWIJOTZSNHNH	3,5-dimethoxy-4-[3,4,5-trihydroxy-6-(hydroxymethyl)-oxan-2-yl]oxybenzoic acid		CA
21	289.0715	$C_{15}H_{14}O_6$	MMONJMOIOLNVKE	2',3,4',6,7-Pentahydroxyflavan		F
22	633.0732	$C_{27}H_{21}O_{18}$	USKARXYQJDURNC	3,6-(HHDP)-glucose		HT
23	635.0890	C_{27} H_{23} O_{18}	MACFXELYCBWKGT	1,2,3-Trigalloyl-O-β-D-glucopyranoside		HT

24	467.0829	$C_{20}H_{20}O_{13}$	DEWITVCPIVXLOM	3-Galloylglucose4-O-(3,4-Dihydroxybenzoyl)		HT
25	479.0821	$C_{21}H_{19}O_{13}$	YUANNBKEZDNSIV	3,3',4',5,6,7-Hexahydroxyflavone-3-O-β-D-Galactopyranoside		F
26	729.1457	$C_{37}H_{29}O_{16}$	DWTOBCBYINHWCP	3′-O-Galloylprocyanidin B5		F
27	631.0936	$C_{28}H_{24}O_{17}$	FOMYLMGOSTVYEE	Myricetin 3-(6-galloylgalactoside)		F
28	449.0722	$C_{20}H_{18}O_{12}$	KJEPOVCGFQFFLL	3,3',4',5,5',7-Hexahydroxyflavone7-O-Arabinoside		F
29	477.1032	$C_{22}H_{22}O_{12}$	LJIXGYLEAVPBHF	4',5,6,7,8-Pentahydroxyisoflavone8-Me ether, 7-O-β-D-glucopyranoside		F
30	479.0829	$C_{21}H_{20}O_{13}$	YUANNBKEZDNSIV	3,3',4',5,6,7-Hexahydroxyflavone-3-O-β-D-Galactopyranoside	Myricetin-3-*O*-β-D-galactopyranoside	F
31	435.0927	$C_{20}H_{20}O_{11}$	PHFGZFOLWQLOEA	Parmentin B		CA
32	449.0717	$C_{20}H_{17}O_{12}$	KJEPOVCGFQFFLL	3,3',4',5,5',7-Hexahydroxyflavone7-O-Arabinoside		F
33	937.0935	$C_{41}H_{30}O_{26}$	WTXYHBLZUNEOJB	2,3,4-Tri-1,6-(S)-HHDP-β-D-glucopyranoside		HT
34	787.1050	$C_{34}H_{28}O_{22}$	XFLTYUCKJRFDOU	1,2,3,4-Tetragalloylglucose	1,2,3,4-Tetragalloylglucopyranoside	HT
35	477.1032	$C_{22}H_{22}O_{12}$	LJIXGYLEAVPBHF	4',5,6,7,8-Pentahydroxyisoflavone8-Me ether, 7-O-β-D-glucopyranoside		F
36	615.0981	$C_{23}H_{18}O_{14}$	AHOPFKRXJRLLGF	3″-O-Galloylmyricitrin		F
37	300.9987	$C_{14}H_6O_8$	AFSDNFLWKVMVRB	Ellagic acid		CA
38	751.0775	$C_{34}H_{24}O_{20}$	JLYPCVMYPPXIDO	Ellagic acid2-O-[3,4,5-Trihydroxybenzoyl-(→4)-α-L-rhamnopyranoside]		CA
39	449.0721	$C_{20}H_{17}O_{12}$	KJEPOVCGFQFFLL	3,3',4',5,5',7-Hexahydroxyflavone7-O-Arabinoside		F
40	197.0452	$C_9H_{10}O_5$	VFPFQHQNJCMNBZ	2,5-Dihidroxy-1,4-benzenedicarboxylic acid	Ethyl gallate	CA
41	389.1085	$C_{16}H_{22}O_{11}$	GOZMBEYOICLJFA	1,2-Propanediol(S)-form1-O-[3,4,5-Trihydroxybenzoyl-(→6)-β-D-glucopyranoside]		CA
42	497.1298	$C_{22}H_{26}O_{13}$	CXAWVDXUHIJTBI	3-O-Caffeoylshikimic acid-4-O-beta-D-Glucopyranoside		CA
43	463.0877	$C_{21}H_{19}O_{12}$	VYOQGRXSRQMRAZ	3,3',4',5,6,7-Hexahydroxyflavone-3-O-α-L-Rhamnopyranoside	Myricetin-3-O-α-L-Rhamnopyranoside	F
44	463.0877	$C_{21}H_{19}O_{12}$	VYOQGRXSRQMRAZ	3,3',4',5,6,7-Hexahydroxyflavone-3-O-α-L-Rhamnopyranoside	Quercetin-3-*O*-β-D-galactopyranoside	F
45	505.0983	$C_{23}H_{22}O_{13}$	LEHONRZMEVUGDI	Isobiflorin-6'-O-(3,4,5-Trihydroxybenzoyl)		Ch
46	599.1039	$C_{28}H_{24}O_{15}$	JFLAOPHOUGDFGC	Quercetin 3-O-[3,4,5-Trihydroxybenzoyl-(→3)-α-L-rhamnopyranoside]		F
47	729.1458	$C_{37}H_{30}O_{16}$	DWTOBCBYINHWCP	3′-O-Galloylprocyanidin B5		F
48	481.0986	$C_{21}H_{22}O_{13}$	OSPSKZAEYSNSGH	3'-(6''-Galloylglucosyl)-phloroacetophenone		CA
49	629.1144	$C_{29}H_{26}O_{16}$	UETPEBBTEWIQAH	3,3',4',5,5',7-Hexahydroxy-2'-methylflavone5'-O-(3,4,5-Trihydroxybenzoyl), 3-O-α-L-rhamnopyranoside		F

50	433.0770	$C_{20}H_{18}O_{11}$	AHCVCOYSTRDXHD	Quercetin-3-D-xyloside		F
51	615.0978	$C_{28}H_{24}O_{16}$	PXGWEUQZDRUMRE	2′′-O-Galloylisoquercitrin		F
52	433.0770	$C_{20}H_{18}O_{11}$	AHCVCOYSTRDXHD	Quercetin-3-D-xyloside	Quercetin-3-*O*-β-D-xylopyranoside	F
53	599.1039	$C_{28}H_{24}O_{15}$	JFLAOPHOUGDFGC	Quercetin 3-O-[3,4,5-Trihydroxybenzoyl-(→3)-α-L-rhamnopyranoside]		F
54	601.0831	$C_{27}H_{22}O_{16}$	DMQBEJUBPKVXEH	3,3',4',5,5',7-Hexahydroxyflavone3-O-[3,4,5-Trihydroxybenzoyl-(→2)-α-L-arabinopyranoside]		F
55	433.0770	$C_{20}H_{18}O_{11}$	AHCVCOYSTRDXHD	Quercetin-3-D-xyloside	Quercetin-3-O-α-L-arabinofuranoside	F
56	629.1142	$C_{29}H_{26}O_{16}$	UETPEBBTEWIQAH	3,3',4',5,5',7-Hexahydroxy-2'-methylflavone5'-O-(3,4,5-Trihydroxybenzoyl), 3-O-α-L-rhamnopyranoside		F
57	447.0927	$C_{21}H_{20}O_{11}$	VKVJRSCYKAOTEB	Quercitrin	Quercetin-3-*O*-α-L-rhamnopyranoside	F
58	477.1032	$C_{22}H_{22}O_{12}$	LJIXGYLEAVPBHF	4',5,6,7,8-Pentahydroxyisoflavone8-Methyl ether, 7-O-β-D-glucopyranoside		F
59	447.0929	$C_{21}H_{20}O_{11}$	VKVJRSCYKAOTEB	3,3',4',7-Tetrahydroxyflavone3-O-β-D-Glucopyranoside		F
60	505.0983	$C_{23}H_{22}O_{13}$	LEHONRZMEVUGDI	Isobiflorin-6'-O-(3,4,5-Trihydroxybenzoyl)		Ch
61	417.0822	$C_{20}H_{18}O_{10}$	POQICXMTUPVZMX	Kaempferol-3-O-alpha-L-arabinoside		F
62	633.0510	$C_{27}H_{21}O_{18}$	USKARXYQJDURNC	3,6-(HHDP)-glucose		HT
63	315.0141	$C_{13}H_{16}O_{9}$	FAARLWTXUUQFSN	3-O-methyl ellagic acid		HT
64	317.0298		YWQCFANDJBOPBN	2',3',5,6,7,8-Hexahydroxyisoflavone		F
65	585.0878	$C_{27}H_{22}O_{15}$	XLHGECRFSYAHQI	Quercetin 3-glycosidesMonosaccharides-3-O-[3,4,5-Trihydroxybenzoyl-(→2)-α-L-arabinopyranoside]	Quercetin-O-(O-galloyl)-pentoside	F
66	615.0986	$C_{28}H_{24}O_{16}$	PXGWEUQZDRUMRE	2′′-O-Galloylisoquercitrin		F
67	431.0978	$C_{21}H_{20}O_{1}$	BEUCMLJKFOWDNP	Kaempferol-3-O-α-L-Rhamnopyranoside		F
68	505.0983	$C_{23}H_{22}O_{13}$	LEHONRZMEVUGDI	Isobiflorin-6'-O-(3,4,5-Trihydroxybenzoyl)		Ch
69	599.1039	$C_{28}H_{24}O_{15}$	JFLAOPHOUGDFGC	3′′-O-Galloylquercitrin		F
70	301.0349	$C_{15}H_{10}O_{7}$	REFJWTPEDVJJIY	Quercetin		F
71	285.0402	$C_{15}H_{10}O_{6}$	LRDGATPGVJTWLJ	3′,4′,5′,7-Tetrahydroxyisoflavone		F
72	315.0508	$C_{16}H_{12}O_{7}$	IZQSVPBOUDKVDZ	3,3′,4′,5-Tetrahydroxy-7-methoxyflavone		F
73	359.0767	$C_{15}H_{20}O_{10}$	SZKFMAOEZUMSRT	3-Acetoxy-4′,5,7-trihydroxy-6-methoxyflavanone		F
74	471.0560	$C_{22}H_{16}O_{12}$	TTWIODVVLGIOKB	3,3',4',5,5',7-Hexahydroxyflavanone(2R,3R)-form4'-O-(3,4,5-Trihydroxybenzoyl)		F

75	577.1350	$C_{30}H_{26}O_{12}$	HIPTUWSXQUYJNF	3,3',4',5,7-Pentahydroxyflavan-(4→8)-3,3',4',5',6-pentahydroxyflavan	F
76	305.0660	$C_{15}H_{14}O_{7}$	JIJWVBHMGUGSCK	Epigallocatechin	F
77	597.1816	$C_{27}H_{34}O_{15}$	FCDASNXDXCOVLC	Hydroxytyrosol 1-glycosides1-O-[4-Hydroxy-3-methoxybenzoyl-(→5)-β-D-apiofuranosyl-(1→2)-β-D-glucopyranoside]	HT
78	751.0781	$C_{34}H_{24}O_{20}$	JLYPCVMYPPXIDO	Ellagic acid2-O-[3,4,5-Trihydroxybenzoyl-(→3)-[3,4,5-trihydroxybenzoyl-(→4)]-α-L-rhamnopyranoside]	HT

List of abbreviations: DNP = Dictionary of Natural Products; GNPS = Global Natural Products Social Networking; F = flavonoid derivatives; CA = carboxylic acid derivatives; Ch = chromone derivatives; HT = hydrolysable tannin derivatives.

11. Correlation analysis of features with meteorological and soil data

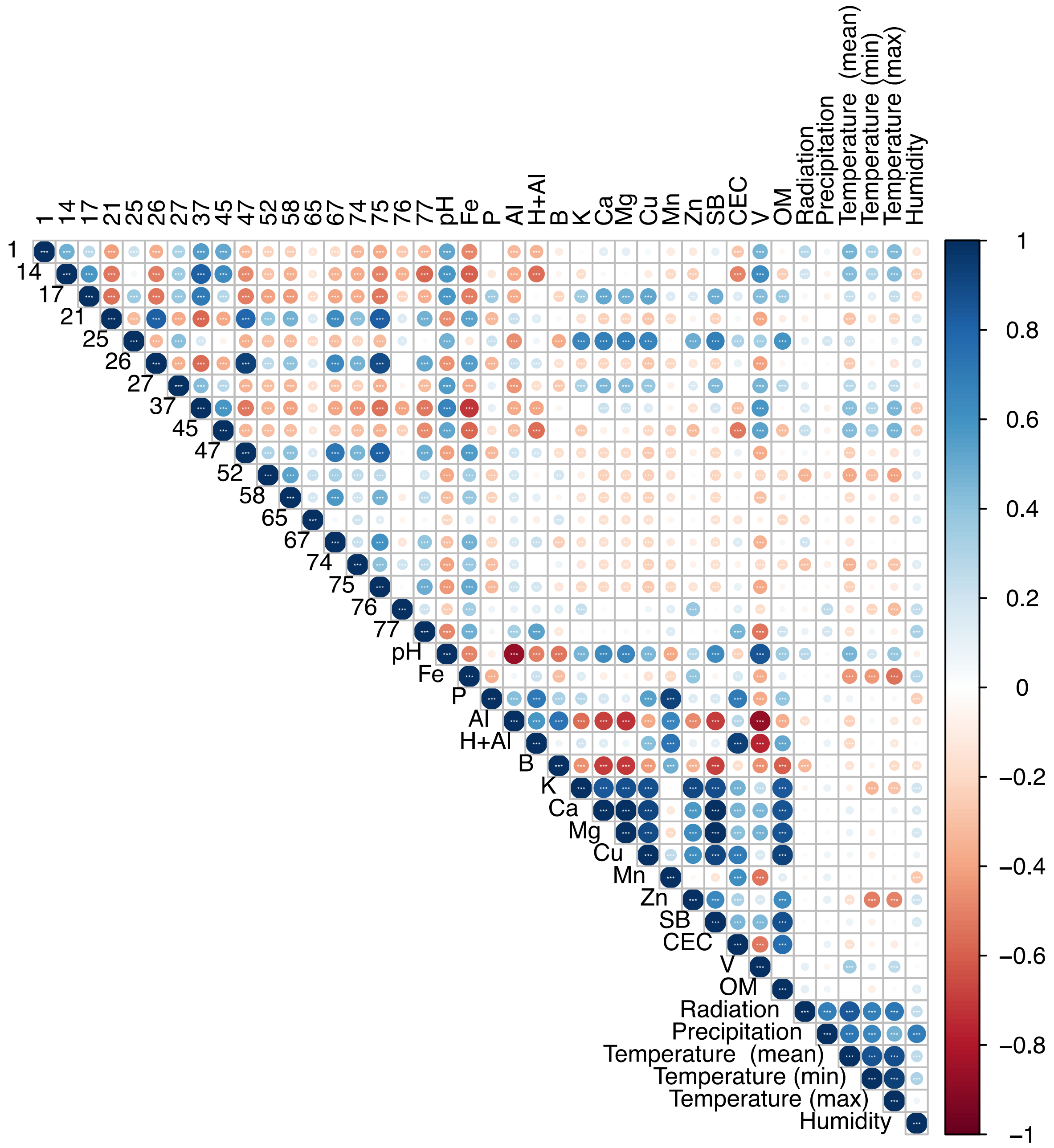

Figure S9. Correlation matrix of features with meteorological and soil data. Fe = Soil iron; Al = Soil aluminum; Mn = Soil manganese; K = Soil potassium; Cu = Soil copper; P = Soil phosphorus; Mg = Soil magnesium; Ca = Soil calcium; Zn = Soil zinc; S.B. = Soil sum of basis; CEC = Soil cation exchange capacity ; OM = Soil organic matter; pH = Soil pH; V = Soil bases saturation; Temperature (mean) = Air mean temperature; Temperature (min) = Air minimum temperature ; Temperature (max) = Air maximum temperature; Humidity = Air relative humidity. * = P<0.001; ** = P<0.01; *** = P<0.05.

11. Variable plot line of selected compounds.

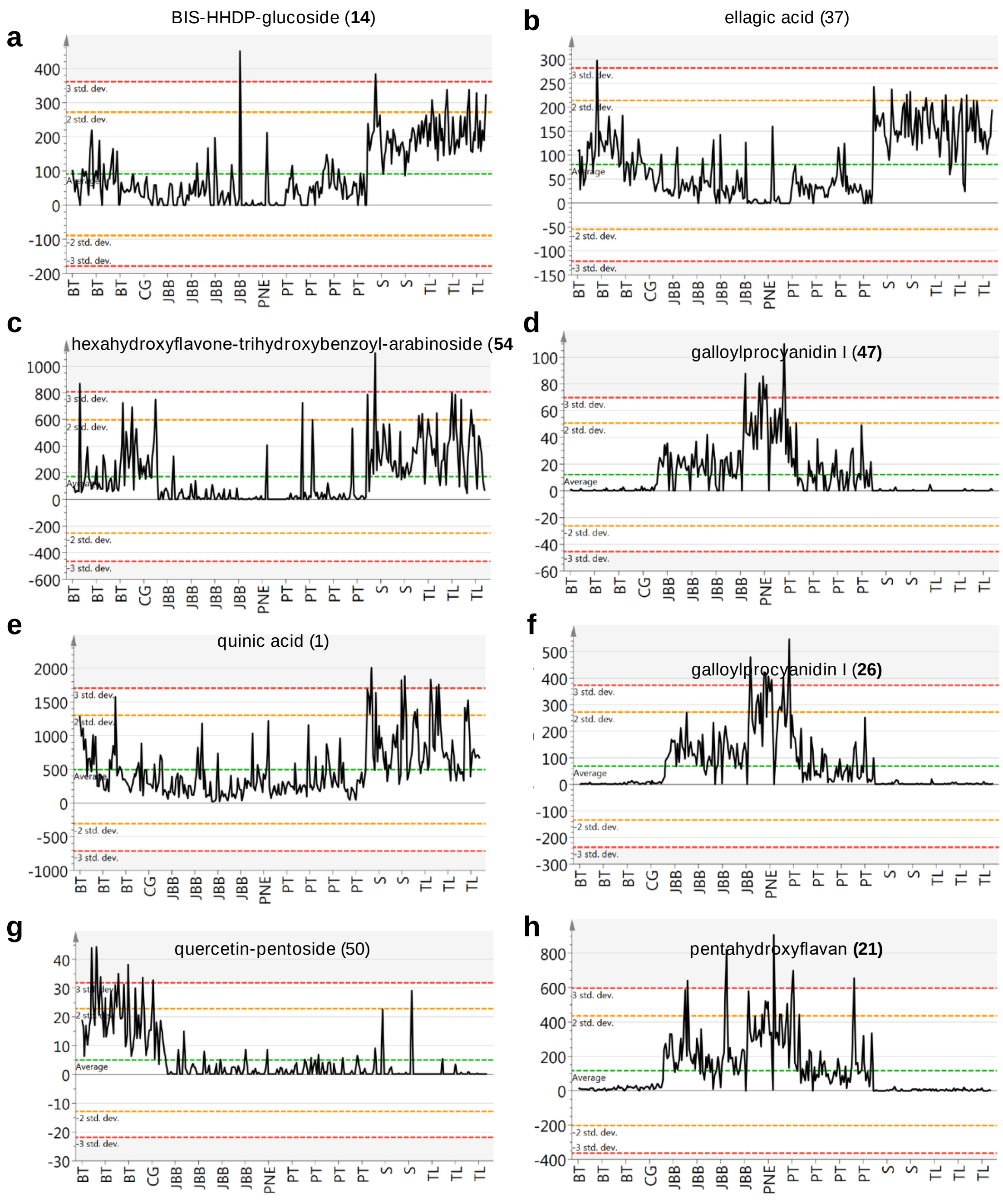

Figure S10. Variable plot line from the MVDA of the selected compounds and their relative intensities for each locality. Variable plot line for (**a**) BIS-HHDP-glucose (14), (**b**) Ellagic acid (37), (**c**) Trihydroxybenzoyl-(→2)-α-L-arabinopyranoside] (54), (**d**) 3′-O-Galloylprocyanidin B5, (e) Quinic acid (1), (**f**) 3′-O-Galloylprocyanidin B5 (26), (**g**) Quercetin-3-D-xyloside (50), (**h**) 2',3,4',6,7-Pentahydroxyflavan (21).

12. Agarose gel electrophoresis of DNA obtained from the leaves of *Myrcia bella*

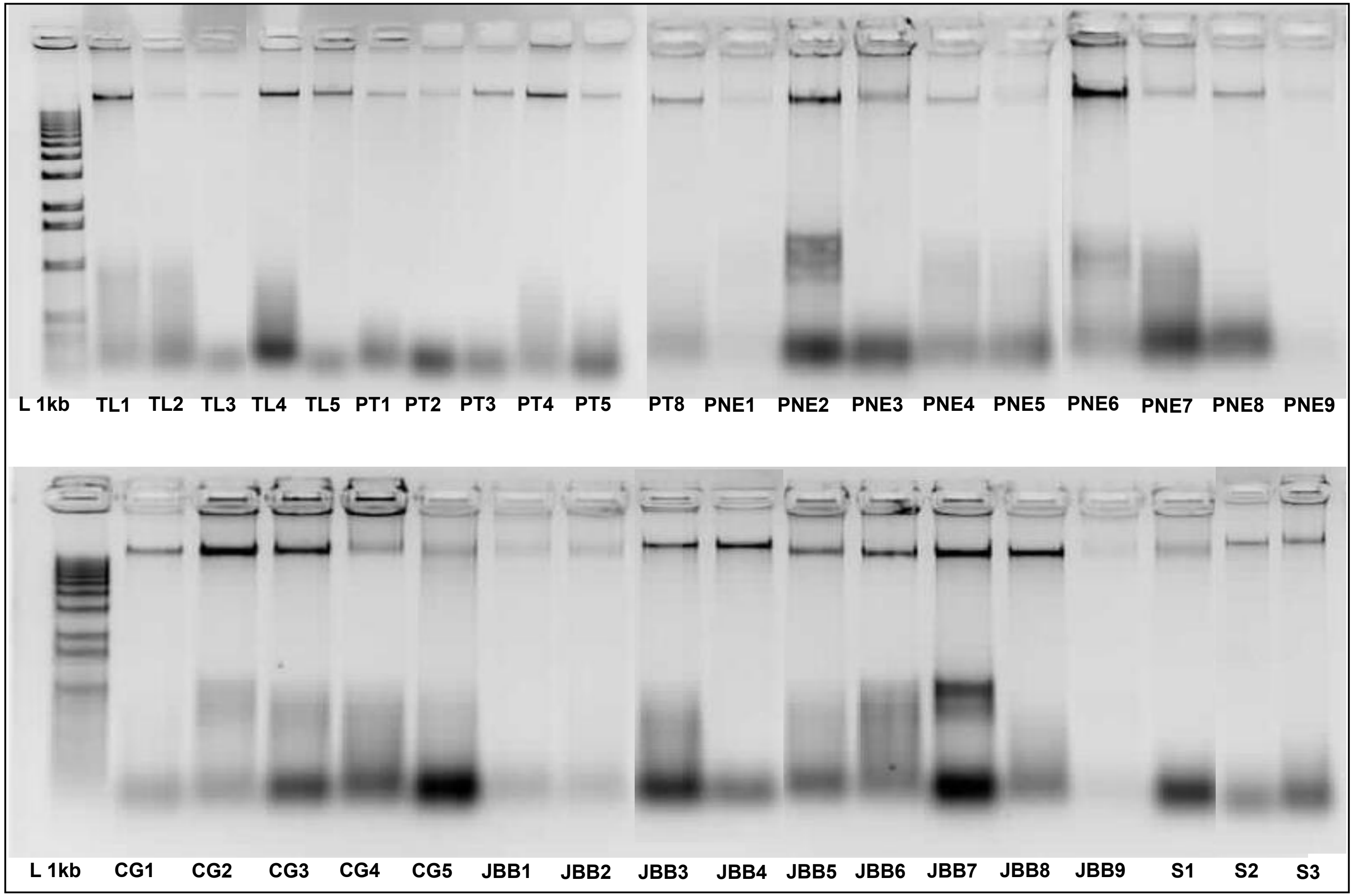

Figure S11. 1,5% agarose gel electrophoresis of DNA obtained from the leaves of specimens of *Myrcia bella* collected in different regions of the Cerrado. List of abbreviations: L 1kB = 50 bp DNA maker; TL = Três Lagoas; PT = Pratânia; PNE = Parque Nacional das Emas; CG = Campo Grande; JBB = Jardim Botânico de Bauru; S = Selvíria.
